# Cyclometalated Iridium(III) Complex–Cationic Peptide Hybrids Trigger Paraptosis in Cancer Cells via an Intracellular Ca^2+^ Overload from the Endoplasmic Reticulum and a Decrease in Mitochondrial Membrane Potential

**DOI:** 10.3390/molecules26227028

**Published:** 2021-11-21

**Authors:** Chandrasekar Balachandran, Kenta Yokoi, Kana Naito, Jebiti Haribabu, Yuichi Tamura, Masakazu Umezawa, Koji Tsuchiya, Toshitada Yoshihara, Seiji Tobita, Shin Aoki

**Affiliations:** 1 Faculty of Pharmaceutical Sciences, Tokyo University of Science, 2641 Yamazaki, Noda, Chiba 278-8510, Japan; balaeri09@gmail.com (C.B.); yokoken4513@gmail.com (K.Y.); 3B17646@alumni.tus.ac.jp (K.N.); haribabusiri970@gmail.com (J.H.); tamura.yuichi@kao.com (Y.T.); 2 Research Institute for Biomedical Sciences (RIBS), Tokyo University of Science, 2641 Yamazaki, Noda, Chiba 278-8510, Japan; 3 Research Institute for Science and Technology (RIST), Tokyo University of Science, 2641 Yamazaki, Noda, Chiba 278-8510, Japan; masa-ume@rs.tus.ac.jp (M.U.); kjtsuchi@rs.tus.ac.jp (K.T.); 4 Department of Chemistry and Chemical Biology, Graduate School of Science and Technology, Gunma University, 1-5-1 Tenjin-cho, Kiryu 376-8515, Japan; yoshihara@gunma-u.ac.jp (T.Y.); tobita@gunma-u.ac.jp (S.T.)

**Keywords:** cyclometalated iridium complex, peptide hybrid, anticancer agents, paraptosis, cytoplasmic vacuolization, Ca^2+^, endoplasmic reticulum

## Abstract

In our previous paper, we reported that amphiphilic Ir complex–peptide hybrids (IPHs) containing basic peptides such as KK(K)GG (K: lysine, G: glycine) (e.g., ASb-2) exhibited potent anticancer activity against Jurkat cells, with the dead cells showing a strong green emission. Our initial mechanistic studies of this cell death suggest that IPHs would bind to the calcium (Ca^2+^)–calmodulin (CaM) complex and induce an overload of intracellular Ca^2+^, resulting in the induction of non-apoptotic programmed cell death. In this work, we conduct a detailed mechanistic study of cell death induced by ASb-2, a typical example of IPHs, and describe how ASb-2 induces paraptotic programmed cell death in a manner similar to that of celastrol, a naturally occurring triterpenoid that is known to function as a paraptosis inducer in cancer cells. It is suggested that ASb-2 (50 µM) induces ER stress and decreases the mitochondrial membrane potential (Δ*Ψ_m_*), thus triggering intracellular signaling pathways and resulting in cytoplasmic vacuolization in Jurkat cells (which is a typical phenomenon of paraptosis), while the change in Δ*Ψ_m_* values is negligibly induced by celastrol and curcumin. Other experimental data imply that both ASb-2 and celastrol induce paraptotic cell death in Jurkat cells, but this induction occurs via different signaling pathways.

## 1. Introduction

Programmed cell death (PCD) is an important pathway controlled by intracellular organelles and is also referred as cellular suicide. PCD is associated with multifunctional biological events such as ontogenesis, the maintenance of tissue homeostasis, and the removal of potentially dangerous cells [1,2]. Two representative categories of PCD are apoptosis and necroptosis, based on intracellular or extracellular signaling pathways, morphological changes, and the activation of specific enzymes [3]. Although numerous antitumor drugs have been developed that induce the apoptosis of cancer, their action is not sufficient for the elimination of cancer cells through typical forms of caspase-dependent PCD.

The induction of non-apoptotic types of PCD such as necroptosis, autophagy, and pyroptosis represents new effective approaches for fighting cancer cells [4,5]. Autophagic cell death has been identified as non-caspase-dependent type of PCD accompanied by plasma membrane blebbing, autophagic vacuoles in the cytoplasm, enlarged organelles, large intracellular vesicles, and the absence of chromatin condensation [6]. Paraptosis has recently been recognized as another non-caspase-dependent type of PCD, as evidenced by the development of endoplasmic reticulum (ER) stress, extensive cytoplasmic vacuolization, and swelling or the dysfunction of the ER and/or mitochondria [7,8,9,10,11]. It has been reported that paraptosis in cancer cells can be induced by viruses [12], metal complexes including copper [13,14,15,16], ruthenium [17,18], titanium [19], iridium [20,21], and rhenium [22], organic compounds such as quinone derivatives [23,24], and naturally occurring compounds such as taxol [25,26], celastrol [27,28], hesperidin [29,30,31], curcumin [32,33,34], gypenoside L [35], morusin [36], chalcomoracin [37], cyclosporine A [38], and ophiobolin A [39]. However, the mechanism responsible for the paraptosis in cancer cells that is induced by these molecules appears to be complicated and has not yet been extensively studied.

In our previous studies, we reported on the design and synthesis of cyclometalated iridium(III) (Ir(III)) complex–peptide hybrids (IPHs) that contain cationic peptides such as a KK(K)GG (K: lysine, G: glycine) sequence that are attached via alkyl chain linkers and which exhibit considerable cytotoxicity against Jurkat cells (human T-lymphocyte cells) by the induction of cellular morphological changes [40,41,42,43,44,45]. It should be noted that cyclometalated Ir(III) complexes and IPHs generally possess excellent chemical and photophysical properties such as high stability in aqueous solution under physiological conditions, high emission quantum yields, and long emission lifetimes in the order of microseconds, generally known as “phosphorescence”. Besides, it was found that IPHs are transferred into living cancer cells to induce their cell death and then exhibit strong green (and yellow) luminescence emission in dead cells, implying that IPHs possess dual functions as an inducer of cell death in Jurkat cells and luminescence probes for dead cells [40,41,42,43,44,45].

**Figure 1:**
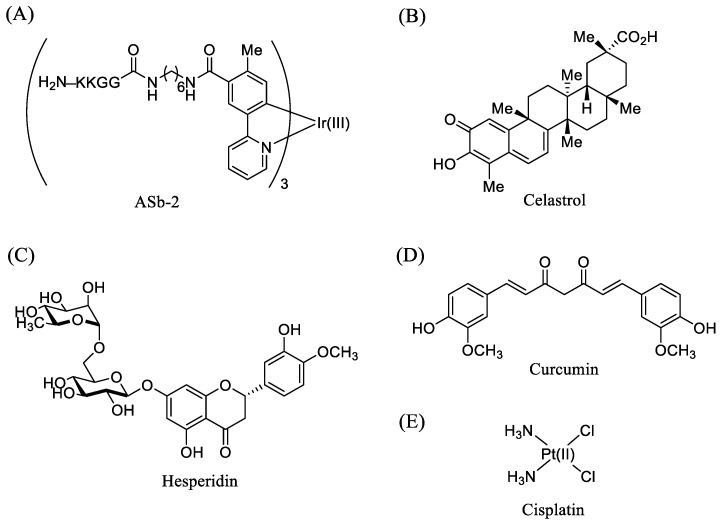
The structures of (**A**) ASb-2, (**B**) celastrol, (**C**) hesperidin, (**D**) curcumin, and (**E**) cisplatin.

For the mechanistic studies of IPH-induced cell death, an IPH containing photoreactive 3-trifluoromethyl-3-phenyldiazirine (TFPD) groups was synthesized to conduct photoaffinity labeling of the target biomolecules of IPHs in Jurkat cells. A proteomic analysis of the products obtained by the photoirradiation of the IPH–TFPD conjugate with Jurkat cells suggested that calmodulin (CaM), a typical intracellular Ca^2+^-binding protein, is one of target proteins of the IPHs. Indeed, ASb-2 (Figure 1A), a typical IPH containing three KKGG peptides that are attached via a C_6_ linker, and its analogs were found to bind to the Ca^2+^–calmodulin (Ca^2+^–CaM) complex, as evidenced by luminescence titrations and so on [41,43]. Our recent mechanistic studies revealed that ASb-2 and its analogs induce an intracellular Ca^2+^ overload possibly via the complexation with the Ca^2+^–CaM complex to activate or inactivate an intracellular Ca^2+^-dependent pathway, eventually resulting in the induction of paraptosis [40,41,42,43,44]. Moreover, we reported the positive relationship between the number of KKKGG peptide (and cationic charges) of IPHs and their cytotoxicity against Jurkat cells [45].

In the present paper, we report on a more detailed mechanistic study of paraptosis in Jurkat cells that is induced by ASb-2 in comparison with the cell death induced by naturally occurring products such as celastrol (a paraptosis inducer), a triterpenoid isolated from *Tripterygium wilfordii* [27,28], hesperidin (a paraptosis inducer), a flavone glucoside present in *Citrus* [29], curcumin (an ER stress and apoptosis inducer), a flavonoid isolated from turmeric yellow rhizome of *Curcuma longa* [32] (Figure 1B–D), and cisplatin (an apotosis inducer, Figure 1E). The relationship of autophagy with cell death induced by ASb-2 is also discussed, as we reported on the upregulation of some marker proteins of autophagy in Jurkat cells treated with other IPHs in our previous publications [42,44].

## 2. Results

### 2.1. Cytotoxic Study of ASb-2, Celastrol, and Hesperidin against Jurkat, HeLaS3, A549, U937, K562, and IMR-90 Cells, as Evaluated by MTT Assay

**Figure 2:**
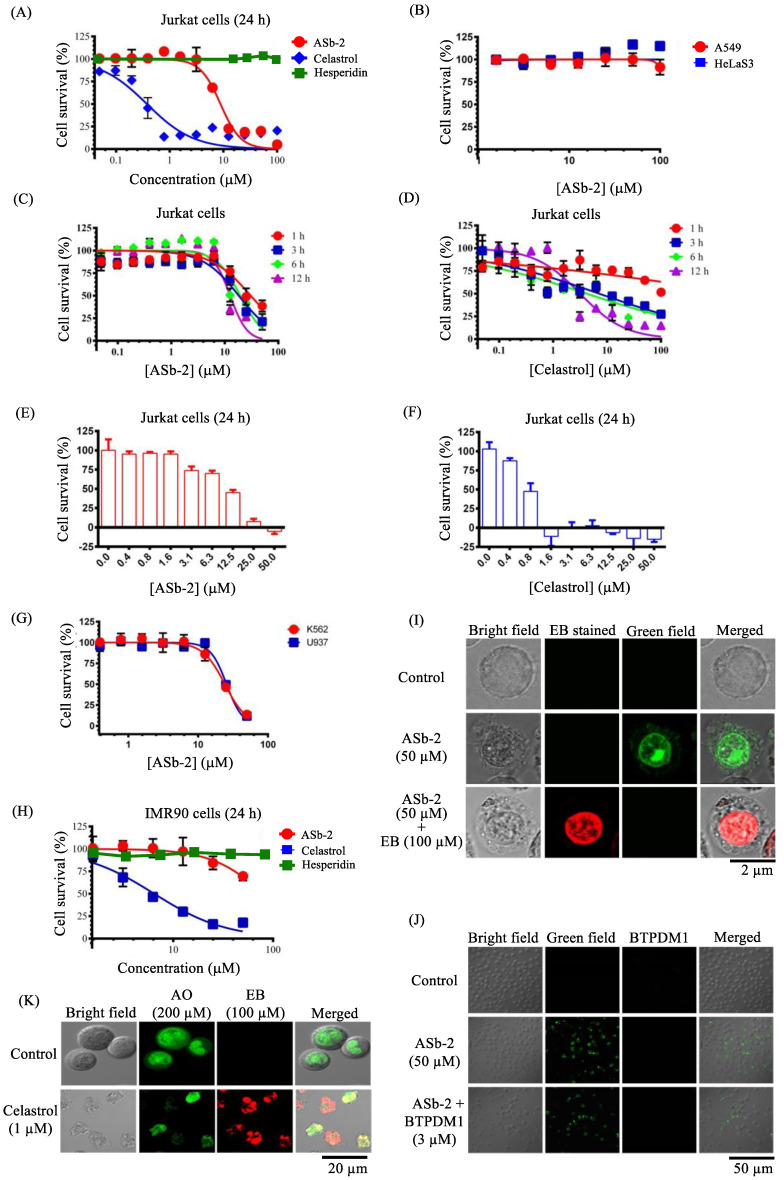
(**A**) The results of MTT assays of Jurkat cells treated with ASb-2 (red spheres), celastrol (blue diamonds), and hesperidin (green squares) for 24 h at 37 °C. (**B**) The results of MTT assays of A549 and HeLaS3 cells treated with ASb-2 (100–1.5 µM) for 24 h at 37 °C. (**C**,**D**) The results of MTT assays of Jurkat cells treated with ASb-2 and celastrol after the incubation for 1, 3, 6, and 12 h at 37 °C. (**E**,**F**) The cell counting results of Trypan blue assays of Jurkat cells treated with AS-2 and celastrol for 24 h at 37 °C. (**G**) The results of MTT assays of K562 and U937 cells treated with ASb-2 for 24 h at 37 °C. (**H**) The results of MTT assays of IMR-90 cells treated with ASb-2, celastrol, and hesperidin for 24 h at 37 °C. (**I**) Typical luminescence confocal microscopic images (Fluoview, FV-1000, Olympus) of Jurkat cells stained with EB (ethidium bromide, 100 µM) in the presence of ASb-2 (50 µM) for 1 h at 37 °C; (**J**) Typical luminescence confocal microscopic images (excitation at 405 nm and emissions at 520–560 nm were used for ASb-2). Excitation at 473 nm and emissions at 680–730 nm were used for BTPDM1) of Jurkat cells stained with BTPDM1 after treatment for 1 h with ASb-2 (50 µM) at 37 °C. (**K**) Typical luminescence confocal microscopic images of Jurkat cells treated with celastrol (1 µM) for 24 h at 37 °C and stained with AO (acridine orange, 200 µM) and EB (ethidium bromide, 100 µM).

The cytotoxic activity of ASb-2 against Jurkat cells was evaluated by an MTT assay (MTT = 3-(4,5-dimethly-2-thiazolyl)-2,5-diphenyl-2*H*-tetrazolium bromide) at different concentrations of ASb-2 (0.05–100 µM) and compared with that of celastrol and hesperidin, which are both reported to induce paraptosis and are commercially available (Figure 2). The IC_50_ values for ASb-2 and celastrol against Jurkat cells after treatment for 24 h were determined to be 8.8 µM and 0.4 µM, respectively, as presented in Figure 2A. As shown in Figure 2B, ASb-2 has negligible cytotoxicity up to 100 µM in A549 (lung) and HeLaS3 (cervical) cells. The IC_50_ values for ASb-2 after incubation for 1 h, 3 h, 6 h, 12 h, and 24 h were 31 µM, 18 µM, 15 µM, 13 µM, and 8.8 µM, respectively, indicating that the cytotoxicity of ASb-2 against Jurkat cells is weakly dependent on the incubation time (Figure 2C). The IC_50_ values for celastrol after the incubation for 1 h, 3 h, 6 h, 12 h, and 24 h were 58 µM, 7.2 µM, 4.2 µM, 3.2 µM, and 0.4 µM, respectively, as presented in Figure 2D, suggesting that celastrol induces cell death more slowly than ASb-2. The cytotoxicity of hesperidin against Jurkat cells was much weaker than that of ASb-2 and celastrol (IC_50_ > 100 µM) (Figure 2A). We then carried out a cell counting assay of Jurkat cells treated with ASb-2 and celastrol using Trypan blue dye, as presented in Figure 2E,F, which indicated that ASb-2 and celastrol induced almost complete cell death at >25 µM and >1.6 µM, respectively, after incubation for 24 h. Next, we examined the cytotoxic effects of ASb-2 on K562 (a human erythroleukemic cell line) and U937 cells (lymphoma cell line). ASb-2 showed moderate cytotoxicity, with IC_50_ values of 24.1 µM and 26.3 µM, respectively, as presented in Figure 2G. For comparison, the IC_50_ values of ASb-2, celastrol, and hesperidin against normal human IMR-90 (human Caucasian fetal lung fibroblast) cells were determined to be >100 µM, 6 µM, and >100 µM, respectively (after incubation for 24 h) (Figure 2H), indicating that ASb-2 exhibits a higher cancer/normal cell selectivity than the other two compounds.

As described in the Introduction, we previously reported that IPHs have dual functions as inducers of PCD and detectors of dead cells based on green luminescence. The term “luminescence” is used in this paper as it includes phosphorescence emissions with longer lifetimes (>microsecond order) and fluorescence emissions with shorter lifetimes (nanosecond order) and it is difficult to distinguish phosphorescence and fluorescence from IPHs and other fluorescent probes used for microscopic observation and flow cytometry measurements in the paper [40,41]. 

Jurkat cells were treated with ASb-2 (50 µM) for 1 h and observed by confocal microscopy in the presence or absence of ethidium bromide (EB) (100 µM), the emission of which is known to increase with the intercalation into double-stranded DNA in dead cells. As shown in Figure 2I, a strong green emission from ASb-2 in dead cells was observed in the absence of EB and a weak green emission was observed in the presence of EB. It was found that the green emission from ASb-2 was very weak in the presence of EB. These phenomena might be explained by the hypothesis that ASb-2 emits a strong emission due to complexation with double-stranded DNA, which is inhibited by EB, although this has not been confirmed in detail.

The question arose as to whether the enhanced green emission from ASb-2 in dead cells was due to low O_2_ levels in dead cells. To examine this further, the molecular probe BTPDM1, which was developed as a versatile O_2_ probe [46,47,48], was used to measure intracellular O_2_ levels in dead cancer cells in the presence or absence of ASb-2. As shown in Figure 2J, a very weak red emission from BTPDM1 (3 µM) was observed before and after the treatment with ASb-2 (50 µM), suggesting that the enhanced emission from ASb-2 in dead Jurkat cells is not due to the low O_2_ levels.

For comparison, morphological changes in Jurkat cells induced by celastrol (1 µM, 24 h) were observed by confocal microscopy, followed by co-staining with AO (acridine orange, 200 µM) (green emission) and EB to confirm that cell death was induced by celastrol (Figure 2K).

### 2.2. Mechanistic Studies of Cell Death Induced by ASb-2 and Celastrol

**Figure 3:**
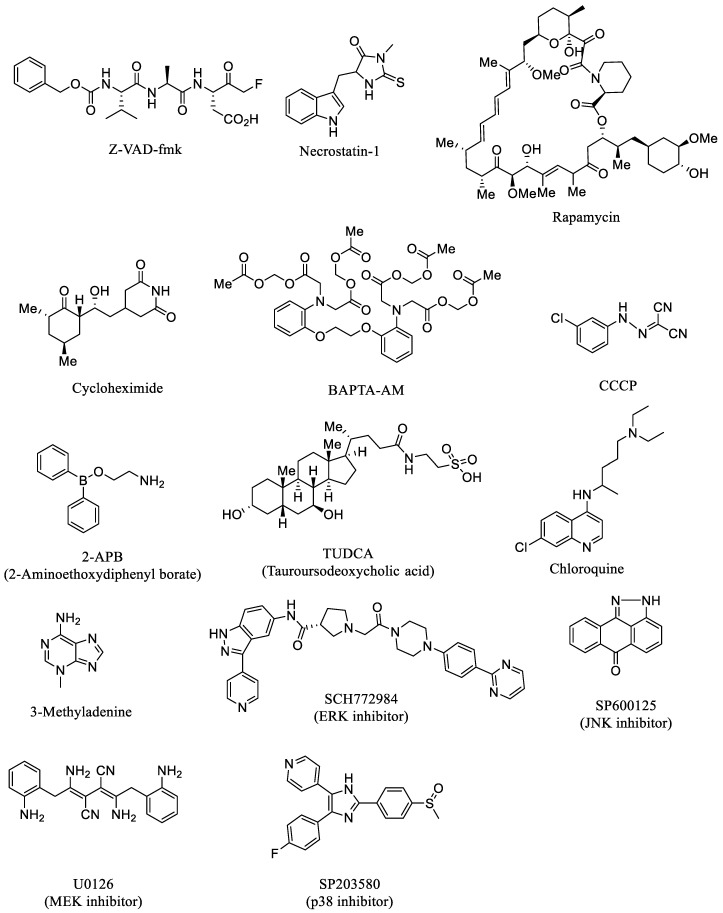
Chemical structures of inhibitors used in this paper.

**Figure 4:**
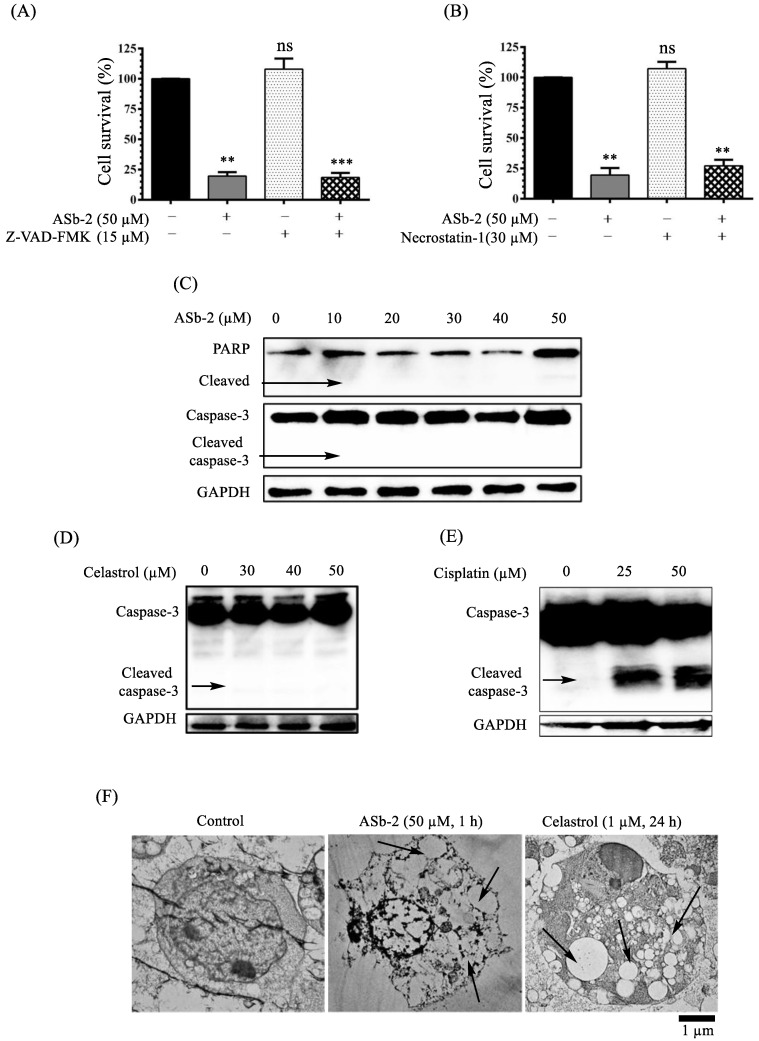
(**A**) The results of MTT assays of Jurkat cells treated with ASb-2 (50 µM) in the presence of Z-VAD-FMK (15 µM) (a broad caspase inhibitor) for 1 h at 37 °C. (**B**) The results of MTT assays of Jurkat cells with ASb-2 in the presence of necrostatin-1(30 µM) (a necroptosis inhibitor) for 1 h at 37 °C. (**C**–**E**) Western blot analysis of apoptosis-related proteins in Jurkat cells treated with ASb-2, celastrol, and cisplatin, respectively. (**F**) TEM images of Jurkat cells after the treatment with ASb-2 (50 µM, 1 h) and celastrol (30 µM, 24 h) at 37 °C. Arrows indicate cytoplasmic vacuolization in Jurkat cells induced by ASb-2 and celastrol, respectively. ** *p* < 0.01 and *** *p* < 0.001 indicate the significant differences from the control. ns: not significant (the statistical data in Figure 4A,B are listed in Table S1 in the Supporting Information).

The mechanism of cell death in Jurkat cells induced by ASb-2 and celastrol was studied by using specific inhibitors of intracellular events. We examined the effect of Z-VAD-FMK (a broad caspase inhibitor) (15 µM) [49] and necrostatin-1 (a necroptosis inhibitor) (30 µM) [50] on the cytotoxicity of ASb-2 (the structures of these inhibitors are shown in Figure 3) and observed that Z-VAD-FMK and necrostain-1 had negligible effects (Figure 4A,B), as we previously reported [40,41,42,43,44,45]. We also carried out Western blot analyses of apoptosis markers such as poly(ADP-ribose) polymerase (PARP) and caspase-3 in Jurkat cells after treatment with ASb-2 and celastrol and observed negligible cleavage of these markers (Figure 4C,D). For comparison, we chose cisplatin, which is known as a clinically approved anticancer agent that induces apoptosis, as a positive control [51] for caspase-3 cleavage and determined its IC_50_ value against Jurkat cells to be 24.3 µM after incubation for 24 h (data not shown) by MTT assay. As shown Figure 4E, caspase-3 cleavage was observed in the presence of cisplatin at 25 and 50 µM for 24 h in Jurkat cells. These data indicate that apoptosis and necroptosis are not the main mechanisms of cell death induced by ASb-2 and celastrol.

We previously reported that IPHs containing a KK(K)GG peptide sequence induce cytoplasmic vacuolization in Jurkat cells, which is one of characteristic phenomena in paraptosis [44,45]. To examine this, Jurkat cells were treated with ASb-2 (50 µM) for 1 h and celastrol (1 µM) for 24 h, embedded in resin, sliced, and stained for transmission electron microscopic (TEM) analysis. Figure 4F shows the cytoplasmic vacuolization of Jurkat cells (indicated by arrows), confirming that paraptosis is caused by ASb-2 and celastrol.

**Figure 5:**
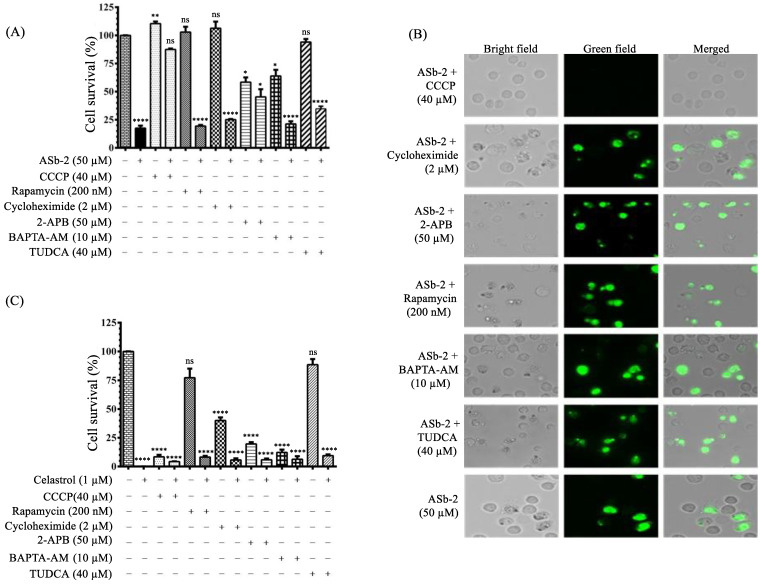
(**A**) The results (cell survival) of MTT assays of Jurkat cells treated with ASb-2 (50 µM) in the presence of CCCP (40 µM), rapamycin (200 nM), cycloheximide (2 µM), 2-APB (50 µM), BAPTA-AM (10 µM), and TUDCA (40 µM) for 1 h at 37 °C. (**B**) Typical microscopy images (Biorevo, BZ-9000, Keyence) of Jurkat cells treated with ASb-2 (50 µM) in the presence of CCCP (40 µM), rapamycin (200 nM), cycloheximide (2 µM), 2-APB (50 µM), BAPTA-AM (10 µM) and TUDCA (40 µM) for 1 h at 37 °C. Scale bar: 20 µm (**C**) The results of MTT assays of Jurkat cells treated with celastrol in the presence of CCCP (40 µM), rapamycin (200 nM), cycloheximide (2 µM), 2-APB (50 µM), BAPTA-AM (10 µM) and TUDCA (40 µM) for 24 h at 37 °C. * *p* < 0.05, ** *p* < 0.01, and **** *p* < 0.0001 indicate significant differences from the control. ns: no significant (the statistical data in Figure 5A,C are listed in Table S2 in the Supporting Information).

We next examined the effect of rapamycin (an mTOR inhibitor), cycloheximide (an inhibitor of protein synthesis), 2-aminoethoxydiphenyl borate (2-APB) (an inhibitor of IP₃ receptors), 1,2-bis(2-aminophenoxy)ethane-*N*,*N*,*N*′,*N*′-tetraacetic acid tetrakis(acetoxymethyl ester) (BAPTA-AM) (a cytoplasmic Ca^2+^ chelator), and tauroursodeoxycholic acid (TUDCA) (a reagent that enhances protein folding and protects cells from ER stress), as well as carbonyl cyanide 3-chlorophenylhydrazone (CCCP) (a mitochondrial uncoupling reagent) [40,43,44]. The structures of these inhibitors are shown in Figure 3. As shown in Figure 5A,B, CCCP (40 µM) inhibited the cell death of Jurkat cells by ASb-2 to a considerable extent, as previously reported [40,43,44]. On the other hand, the cell death induced by celastrol (1 µM, 24 h) was not inhibited by CCCP (40 µM), rapamycin (200 nM), cycloheximide (2 µM), 2-APB (50 µM), BAPTA-AM (10 µM), and TUDCA (40 µM) (Figure 5C). It should be noted that CCCP, cycloheximide, 2-APB, and BAPTA-AM [52] alone exhibited negligible cytotoxicity against Jurkat cells after incubation for 1 h, while these reagents slowly induced cell death (after incubation for 24 h) up to 92%, 60%, 81%, and 88%, respectively.

**Figure 6:**
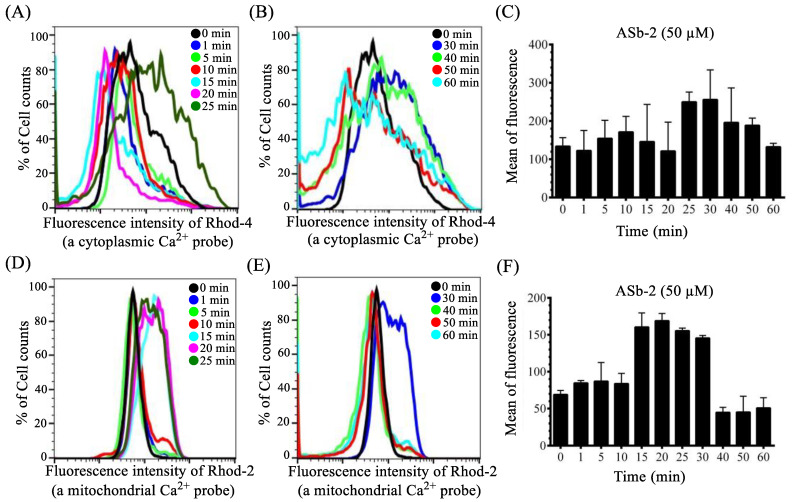

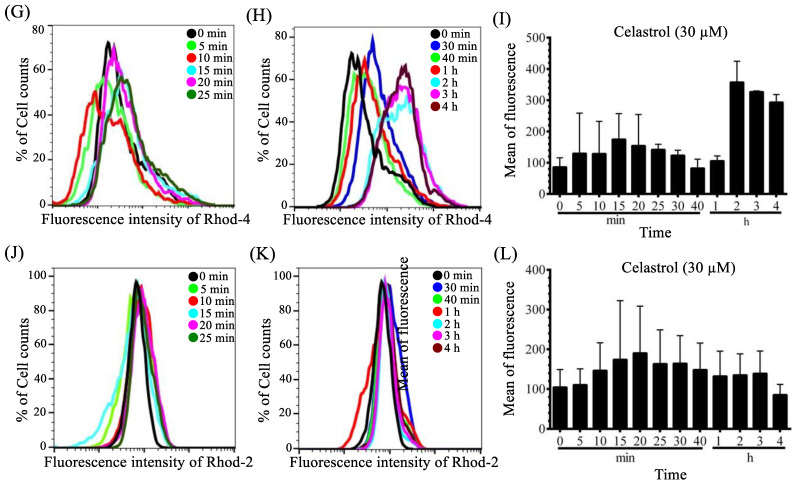
(**A**–**C**) Flow cytometric analysis of Jurkat cells stained with Rhod-4/AM (5 µM), followed by treatment with ASb-2 (50 µM) at 37 °C. (**D**–**F**) Flow cytometric analysis of Jurkat cells stained with Rhod-2/AM (5 µM), followed by treatment with ASb-2 (50 µM) at 37 °C. (**G**–**I**) Flow cytometric analysis of Jurkat cells that had been treated with celastrol (30 µM) and stained with Rhod-4/AM (5 µM) at 37 °C. (**J**–**L**) Flow cytometric analysis of Jurkat cells that had been treated with celastrol (30 µM) and stained with Rhod-2/AM (5 µM) at 37 °C.

**Figure 7:**
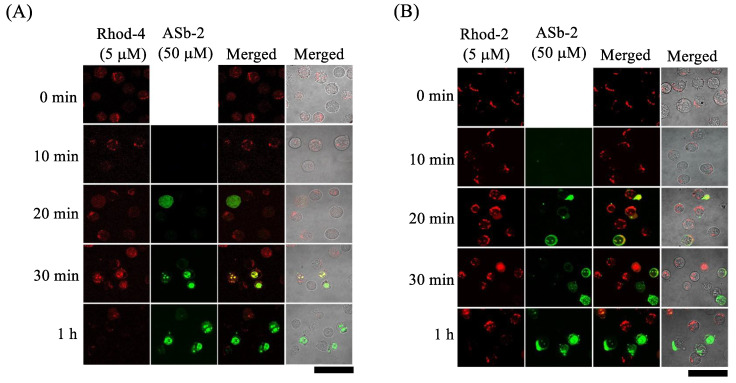

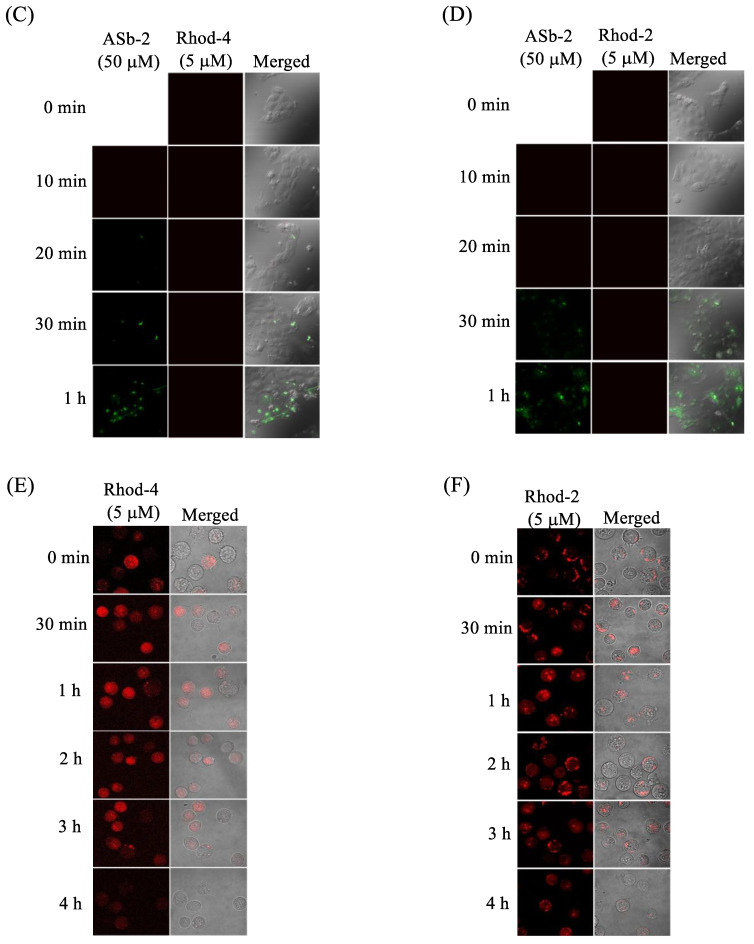
(**A**) Typical luminescence confocal microscopic images (Fluoview, FV-1000, Olympus) of Jurkat cells treated with ASb-2 (50 µM) and then stained with Rhod-4/AM (5 µM) at 37 °C. (**B**) Typical confocal microscopic images of Jurkat cells treated with ASb-2 (50 µM) and then stained with Rhod-2/AM (5 µM) at 37 °C. (**C**) Typical luminescence confocal microscopic images (Fluoview, FV-1000, Olympus) of IMR-90 cells treated with ASb-2 (50 µM) and then stained with Rhod-4/AM (5 µM) at 37 °C. (**D**) Typical confocal microscopic images of IMR-90 cells treated with ASb-2 (50 µM) and then stained with Rhod-2/AM (5 µM) at 37 °C. (**E**) Typical luminescence confocal microscopic images of Jurkat cells treated with celastrol (30 µM) and then stained with Rhod-4/AM (5 µM) at 37 °C. (**F**) Typical confocal microscopic images of Jurkat cells treated with celastrol (30 µM) and then stained with Rhod-2/AM (5 µM) at 37 °C. Scale bar: 25 µm.

In our previous study, we reported that cytosolic Ca^2+^ overload is induced by ASb-2 in the process of the cell death in Jurkat cells [40]. In the present work, we measured time-dependent Ca^2+^ concentrations in the cytoplasm and mitochondria of Jurkat and IMR-90 cells, which had been treated with ASb-2 and celastrol, by using Rhod-4/AM (a cytoplasmic Ca^2+^ probe) [44] and Rhod-2/AM (a mitochondrial Ca^2+^ probe) [53] (Figure 6 and Figure 7). Figure 6A–C indicates that ASb-2 induces a considerable increase in the Ca^2+^ concentrations in the cytoplasm after incubation for 25–40 min, and Figure 6D–F indicates that ASb-2 also induces a considerable increase in the Ca^2+^ concentrations in the mitochondria after incubation for 15–30 min, suggesting that the enhancement of the mitochondrial Ca^2+^ concentrations is more rapid than that of the cytoplasmic Ca^2+^ concentrations (Figure 6F vs. Figure 6C). The confocal microscopic images in Figure 7A,B indicate that ASb-2 increases Ca^2+^ concentrations in the cytoplasm and mitochondria in 20–30 min. On the other hand, 2–4 h are required to achieve a cytoplasmic Ca^2+^ overload by celastrol (Figure 6G–I) and the mitochondrial Ca^2+^ concentration changed negligibly (Figure 6J–L) (see also Figure 7E,F). We then measured time-dependent Ca^2+^ concentrations in IMR-90 cells using Rhod-4/AM and Rhod-2/AM. Figure 7C,D indicates that ASb-2 barely increases the Ca^2+^ concentrations in the cytoplasm and mitochondria after incubation for 10–60 min, and shows very weak green emission in IMR-90 cells.

**Figure 8:**
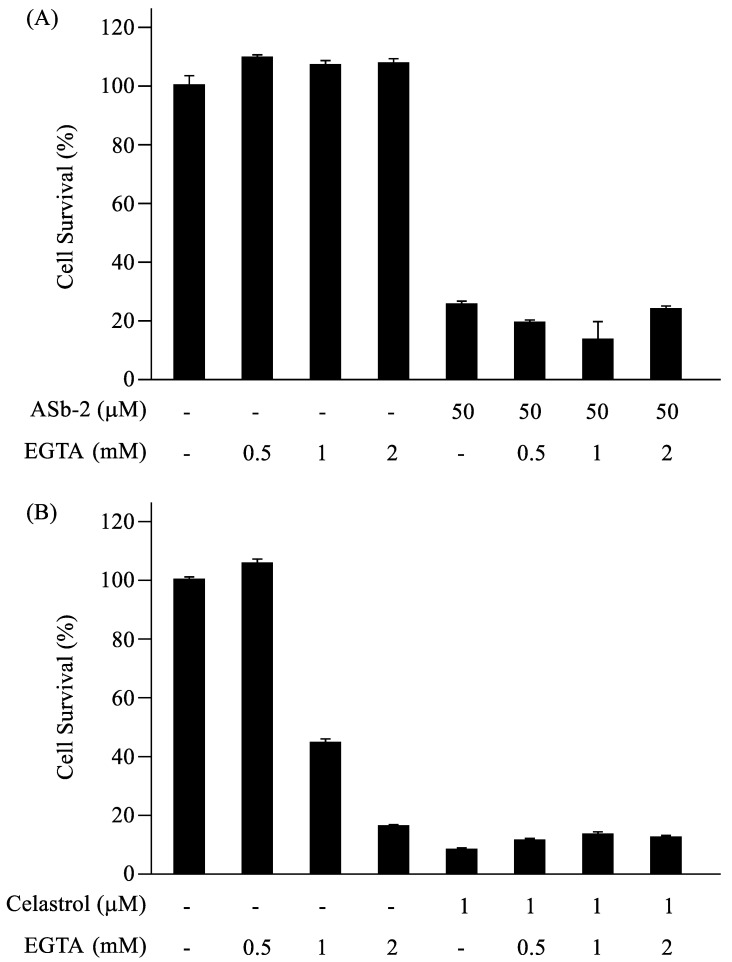
(**A**) The results of MTT assays of Jurkat cells treated with ASb-2 (50 µM) in the presence of EGTA (0.5, 1, and 2 mM) for 1 h at 37 °C. (**B**) The results of MTT assays of Jurkat cells treated with celastrol (1 µM) in the presence of EGTA (0.5, 1, and 2 mM) for 24 h at 37 °C.

We carried out the MTT assays of Jurkat cells with ASb-2 and celastrol in the presence of ethylene glycol-bis(*β*-aminoethylether)-*N,N,N*′*,N*′-tetraacetic acid (EGTA) (0.5, 1, and 2 mM), a Ca^2+^ chelator, in the incubation medium to check the induction of Ca^2+^ transfer from the incubation medium to cytoplasm by ASb-2 and celastrol. However, a negligible effect of EGTA on the cytotoxicity of ASb-2 and celastrol was observed, as shown in Figure 8. These results suggest that the Ca^2+^ influx into mitochondria proceeds inside the cell rather than via the Ca^2+^ transport from outside the cell.

**Figure 9:**
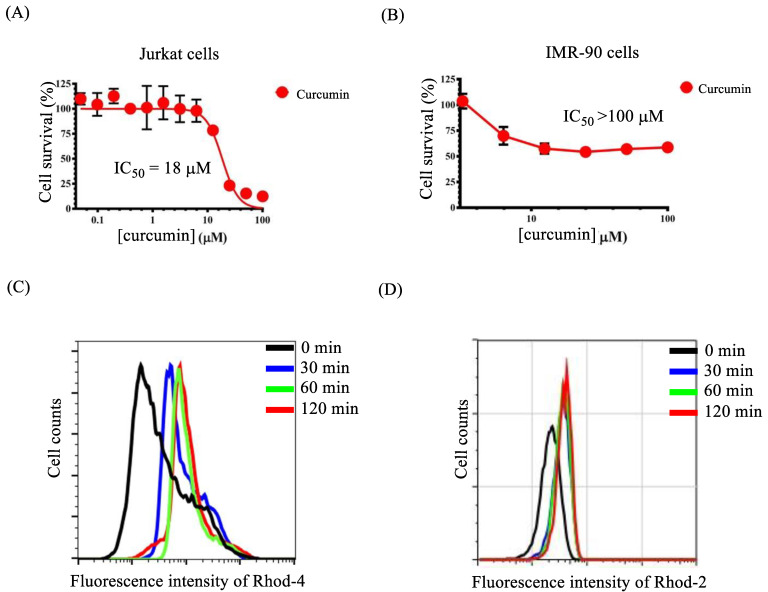
(**A**) The results of MTT assays of Jurkat cells treated with curcumin for 24 h at 37 °C. (**B**) The results of MTT assays of IMR-90 cells treated with curcumin for 24 h at 37 °C. (**C**) Flow cytometry analysis of Jurkat cells treated with curcumin (50 µM) and then stained with Rhod-4/AM (5 µM) for 30, 60, and 120 min at 37 °C. (**D**) Flow cytometry analysis of Jurkat cells treated with curcumin (50 µM) and then stained with Rhod-2/AM (5 µM) for 30, 60, and 120 min at 37 °C.

The changes in intracellular Ca^2+^ concentrations induced by ASb-2 and celastrol were compared using a treatment with curcumin, a compound that is well known to induce apoptosis, with IC_50_ values of 18 µM and >100 µM against Jurkat and IMR-90 cells, respectively (Figure 9A,B) [54]. It was found that curcumin induces cytosolic and mitochondrial Ca^2+^ overload after incubation for 30–120 min, as shown in Figure 9C,D.

### 2.3. Measurement of Mitochondrial Membrane Potential (ΔΨ_m_)

**Figure 10:**
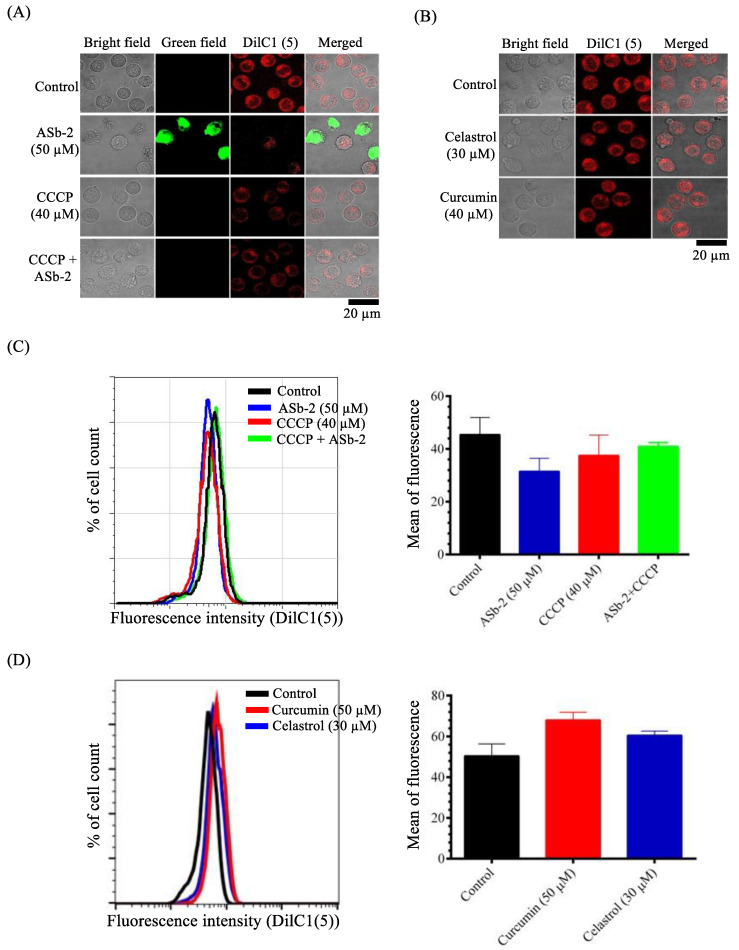
Measurement of Δ*Ψ_m_* in Jurkat cells after treatment with ASb-2 (50 µM), celastrol (30 µM), and curcumin (50 µM) in Jurkat cells stained with DilC1(5) (500 nM), as a cationic cyanine dye. (**A**,**B**) Luminescence confocal microscopic studies of Δ*Ψ_m_* in Jurkat cells in the presence of ASb-2 (50 µM), celastrol (30 µM), and curcumin (50 µM) for 1 h at 37 °C with co-staining of DilC1(5) (500 nM). (**C**,**D**) Flow cytometry analysis of Δ*Ψ_m_* in Jurkat cells in the presence of ASb-2 (50 µM), curcumin (50 µM), and celastrol (30 µM) for 1 h at 37 °C with co-staining with DilC1(5) (500 nM).

The mitochondrial membrane potential (Δ*Ψ_m_*) of Jurkat cells was measured in the presence of ASb-2, celastrol (a paraptosis inducer), and curcumin (an apoptosis inducer). Jurkat cells were pre-treated with or without CCCP (40 µM), which is known as an uncoupling reagent and an inhibitor of the ASb-2-induced cell death (Figure 5A,B), for 1 h, and then treated with ASb-2 (50 µM), celastrol (30 µM), or curcumin (50 µM) for 1 h, after which the Δ*Ψ_m_* values were measured by using DilC1(5) (500 nM) by confocal microscopy and flow cytometry. The confocal microscopic images and flow cytometric analysis shown in Figure 10A,C suggest that the Δ*Ψ_m_* values are decreased by ASb-2 (50 µM) and restored in the presence of CCCP (40 µM). These results imply that CCCP cancels Ca^2+^ overload by compromising the membrane potential of mitochondria [40,43,44] and the decrease in Δ*Ψ_m_* induced by ASb-2. Interestingly, a small increase in Δ*Ψ_m_* was observed by the treatment with celastrol and curcumin, as shown in Figure 10B,D, although the details are yet to be studied.

### 2.4. Relationship of Autophagy with Paraptosis Induced by ASb-2 and Celastrol

**Figure 11:**
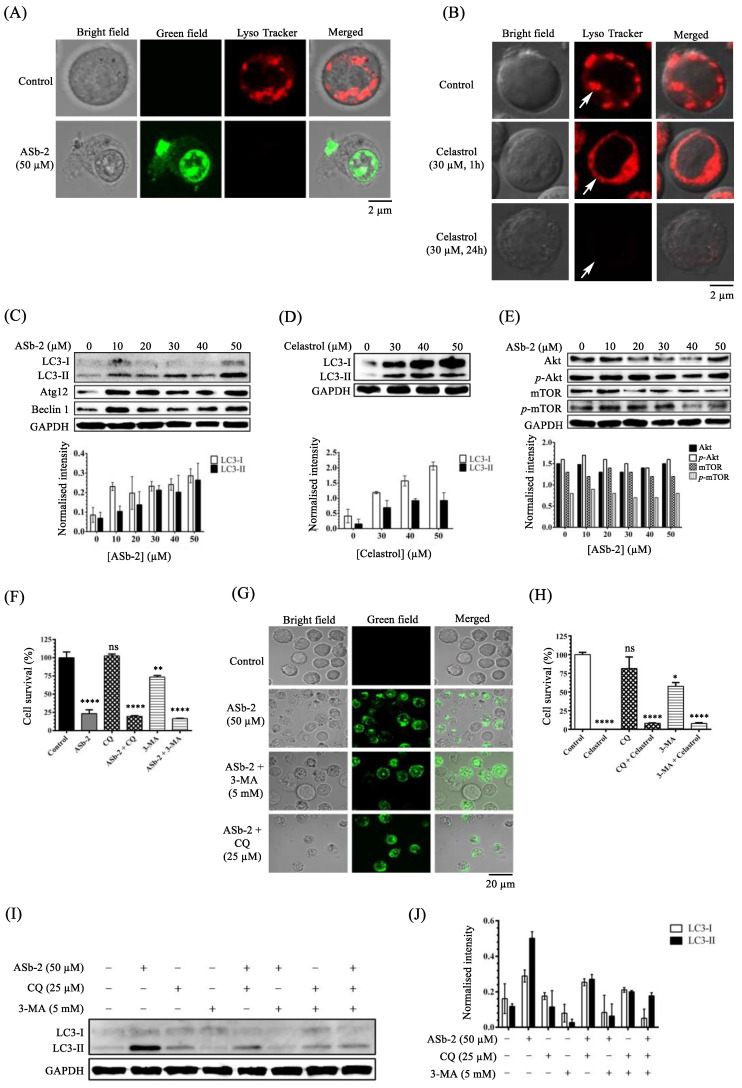
(**A**,**B**) Typical luminescence confocal microscopic images (Fluoview, FV-1000, Olympus) of Jurkat cells stained with LysoTracker red in the presence of ASb-2 (50 µM, 1 h) and celastrol (30 µM, 1 or 24 h) at 37 °C. (**C**) Autophagy markers such as LC3-I/II, Atg12, and beclin-1 were confirmed by Western blot analyses in Jurkat cells for 1 h at 37 °C. (**D**) LC3-I/II expression levels were confirmed by Western blot analyses in the presence or absence of celastrol in Jurkat cells for 1 h at 37 °C. (**E**) Western blot analyses in Akt/mTOR signaling pathway. (**F**) The results of MTT assays of Jurkat cells treated with ASb-2 (50 µM) in the presence of CQ (25 µM) and 3-MA (5 mM) for 1 h at 37 °C. (**G**) Typical microscopy images (Biorevo, BZ-9000, Keyence) of Jurkat cells treated with ASb-2 (50 µM) in the presence of 3-MA (5 mM) and CQ (25 µM) for 1 h at 37 °C. (**H**) The results of MTT assays of Jurkat cells that had been treated with celastrol (1 µM) in the presence of CQ (25 µM) and 3-MA (5 mM) for 24 h at 37 °C. (**I**,**J**) The results of Western blot analyses of LC3-I/II in Jurkat cells treated with ASb-2 in the presence or absence of CQ and 3-MA for 1 h at 37 °C. * *p* < 0.05, ** *p* < 0.01, and **** *p* < 0.0001 indicate the significant differences from the control. ns: not significant (the statistical data in Figure 11F,H are listed in Table S3 in the Supporting Information).

It has been reported that paraptosis is frequently accompanied by autophagy [31], which is regulated by a series of signaling networks such as PI3k/Akt, AMPK, MAPK/ERK1/2, and mTORC1 pathways, and lysosomal degradation is also observed as a characteristic morphological change that occurs in autophagy [55]. The structures of lysosomes were observed by co-staining Jurkat cells with LysoTracker Red in the presence of ASb-2 (50 µM) and celastrol (30 µM). As shown in Figure 11A,B, the emission of LysoTracker Red in lysosomes (indicated with white arrows) was negligible in the presence of ASb-2 (after incubation for 1 h) and celastrol (after incubation for 24 h), indicating that lysosomes are degraded by these two agents.

We then examined autophagy markers such as LC3-I/II, Atg12, and Beclin-1 by Western blot analyses, and the results showed a substantial upregulation of LC3-I/II, Atg12, and Beclin-1 by ASb-2 and celastrol and that this upregulation was dose-dependent (Figure 11C,D). The Akt signaling network is frequently hyperactivated in many human cancer cells [56] and mTOR is a downstream effector that is involved in various signaling pathways and is automatically activated during certain cellular processes such as proliferation, growth, protein synthesis, transcription, ribosomal biogenesis, and cytoskeletal organization. In addition, mTOR is dysregulated in many types of cancers and in type-2 diabetes [57]. However, ASb-2 was found to have a negligible effect on the Akt/mTOR signaling pathway, as shown in Figure 11E, and rapamycin, a mTOR inhibitor, had a negligible effect on the cell death, as shown in Figure 5A. The results of Western blot analyses suggest that LC3-II is upregulated by ASb-2 (Figure 11F,G) and this effect is cancelled by chloroquine (CQ) (an inhibitor of lysosomal function) [58] and 3-methyladenine (3-MA) (an inhibitor of autophagosomal formation) [59], whose structures are shown in Figure 3 (treated for 1 h). The fact that CQ and 3-MA negligibly inhibited the cell death, as shown in Figure 11H–J, suggests that autophagy is not so important in paraptosis that is induced by ASb-2 and celastrol, while the expression of autophagy-related molecules is downregulated by these two compounds.

### 2.5. Effect of ASb-2 and Celastrol on the MAPK Signaling Pathway

**Figure 12:**
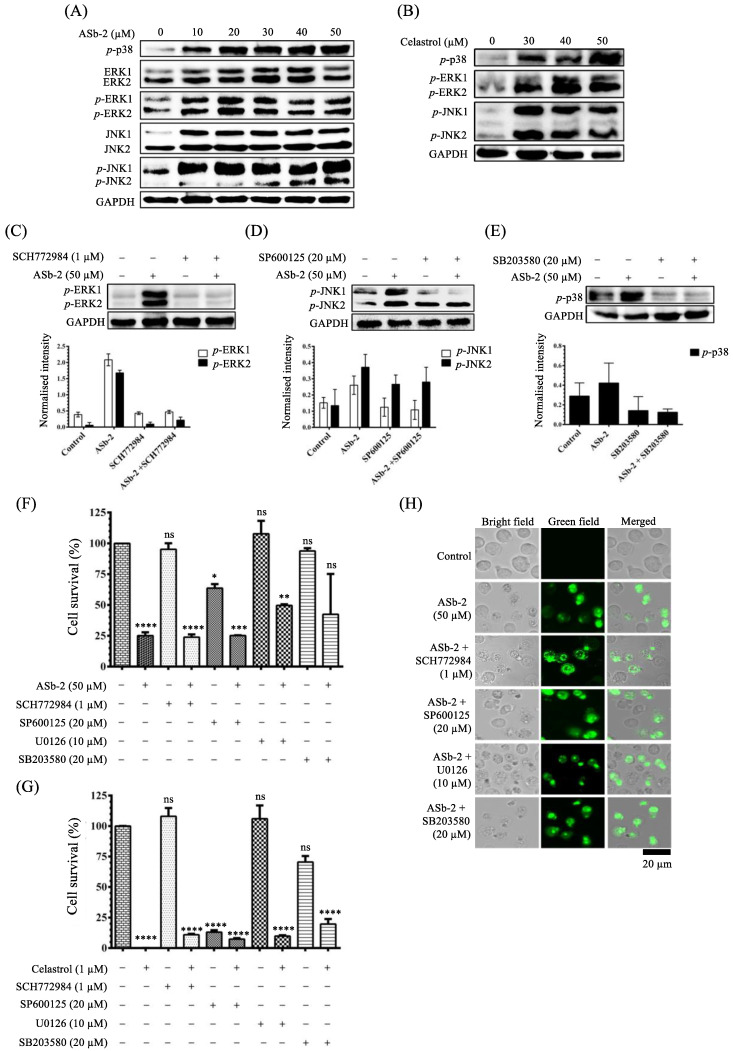
(**A**) Western blot analyses of the MAPK signaling pathway in the presence of ASb-2 in Jurkat cells for 1 h at 37 °C. **(B**) Western blot analyses of the MAPK signaling pathway in the presence of celastrol in Jurkat cells for 1 h at 37 °C. (**C)** The results for the *p*-ERK1/2 expression in Jurkat cells treated with ASb-2 in the presence of SCH772984 for 1 h at 37 °C. (**D**) The results of *p*-JNK1/2 expression of Jurkat cells treated with ASb-2 in the presence of SP600125 for 1 h at 37 °C. (**E**) The results of *p*-p38 expression of Jurkat cells treated with ASb-2 in the presence of SB203580 for 1 h at 37 °C. (**F**) The results of MTT assays of Jurkat cells that had been treated with ASb-2 in the presence of SCH772984, SP600125, U0126, and SB203580 for 1 h at 37 °C. (**G**) The results of MTT assay of Jurkat cells treated with celastrol in the presence of SCH772984, SP600125, U0126, and SB203580 for 24 h at 37 °C. (**H**) Typical microscopy images (Biorevo, BZ-9000, Keyence) of Jurkat cells treated with ASb-2 (50 µM) in the presence of SCH772984, SP600125, U0126, and SB203580 for 1 h at 37 °C. * *p* < 0.05, ** *p* < 0.01, *** *p* < 0.001 and **** *p* < 0.0001 indicate the significant differences from the control. ns: not significant (the statistical data in Figure 12F,G are listed in Table S4 in the Supporting Information).

We next examined the role of the MAPK signaling pathway, which is typically regulated by extracellular signal-regulated kinases 1/2 (ERK1/2), c-Jun amino(N)-terminal kinases 1/2/3 (JNK1/2/3), p38 isoforms (*α*, *β*, *γ*, and Δ), and ERK5, in the ASb-2-induced paraptosis. It is known that the MAPK signaling pathway is involved in the induction of autophagy [60,61,62]. The results of Western blot analyses of marker proteins of the MAPK signaling pathway shown in Figure 12A,B suggest that the phosphorylation of p38, ERK1/2, and SAPK/JNK is enhanced by ASb-2 and celastrol and that this enhancement is concentration-dependent. The upregulation of *p*-ERK1/2, SAPK/JNK, and *p*-p38 by ASb-2 was substantially inhibited by SCH772984 (an ERK inhibitor) (1 µM), SP600125 (a JNK inhibitor) (20 µM), and SB203580 (a p38 inhibitor) (20 µM) (Figure 12C–E) (the chemical structures of these inhibitors are shown in Figure 3). However, the effect of SCH772984, SP600125, U0126 (a MEK 1/2 inhibitor) (10 µM), and SB203580 on cell death was negligible (Figure 12F–H). These data allowed us to conclude that MAPK is activated by ASb-2 and celastrol, although these effects are not critical in the paraptosis induced by these two compounds.

### 2.6. Relationship between ASb-2—Induced Cell Death and ER Stress in Jurkat Cells

**Figure 13:**
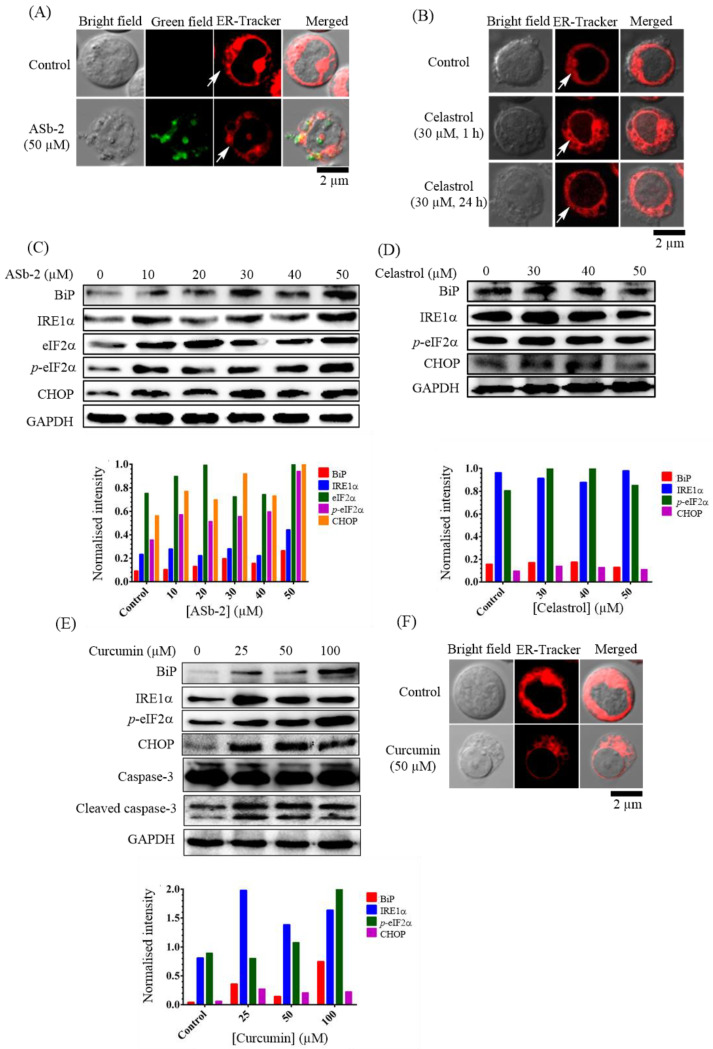
(**A**) Typical luminescence confocal microscopic images (Fluoview, FV-1000, Olympus) of Jurkat cells stained with ER-Tracker Red in the presence of ASb-2 (50 µM) for 1 h at 37 °C. (**B**) Typical confocal microscopic images (Fluoview, FV-1000, Olympus) of Jurkat cells stained with ER-Tracker Red in the presence of celastrol (30 µM) for 1 or 24 h at 37 °C. (**C**) Western blot analyses of ER stress markers such as BiP, IRE1α, eIF2α, *p*-eIF2α, and CHOP in the presence or absence of ASb-2 at various concentrations in Jurkat cells for 1 h at 37 °C. (**D**) The expression levels of BiP, IRE1α, *p*-eIF2α, and CHOP were confirmed by Western blot analyses in the presence or absence of celastrol at various concentrations in Jurkat cells for 1 h at 37 °C. (**E**) The expression levels of BiP, IRE1α, *p*-eIF2α, and CHOP were confirmed by Western blot analyses in the presence or absence of curcumin at various concentrations in Jurkat cells for 1 h at 37 °C. (**F**) Typical luminescence confocal microscopic images (Fluoview, FV-1000, Olympus) of Jurkat cells stained with ER-Tracker Red in the presence of curcumin (50 µM) for 1 h at 37 °C.

In the present study, the involvement of ER stress in the cell death caused by ASb-2 and celastrol was examined by using ER-Tracker Red on confocal microscopy and Western blot analysis. The confocal microscopic observation shows the decrease in red emission from ER-Tracker after incubation with ASb-2 (50 µM) (Figure 13A), while the emission of ER-Tracker Red was observed after incubation with celastrol (30 µM) for 1 h and 24 h (white arrows in Figure 13B). We then conducted Western blot analyses to evaluate the ER functions using BiP, IRE1α, eIF2α, *p*-eIF2α, and CHOP proteins. As shown in Figure 13C,D, the upregulation of BiP, IRE1α, eIF2α, *p*-eIf2α, and CHOP was observed in the presence of ASb-2, and a negligible change in the expression levels of BiP, IRE1α, eIf2α, *p*-eIF2α, and CHOP was observed in the presence of celastrol. BiP is a chaperone that binds to unfolded protein response (UPR) sensors such as IRE1 and PERK, which are tightly regulated under hemostatic and ER stress conditions. It has been described that these UPR sensors play key roles in the detection and restoration of unfolded or misfolded proteins [63]. IRE1α is activated by the release of ER chaperone BiP from its luminal domain [64], while eIF2α is phosphorylated by PERK, resulting in the inhibition of the protein synthesis to reduce the amount and/or number of unfolded proteins. The activation of PERK leads to the upregulation of C/EBP homologous protein (CHOP), a transcription factor, during ER stress [65]. This information, together with the results of our experiments, suggests that the upregulation of BiP and UPR sensors is due to the ER stress induced by ASb-2 in Jurkat cells. However, the activation mechanisms of the three proteins BiP, IRE1α, and eIF2α are unclear [66]. In contrast, curcumin, an apoptosis inducer, induced the cleavage of caspase-3 (Figure 13E), the upregulation of BiP, IRE1α, *p*-eIF2α, and CHOP (Figure 13E), and a decrease in ER-Tracker emission (Figure 13F).

**Figure 14:**
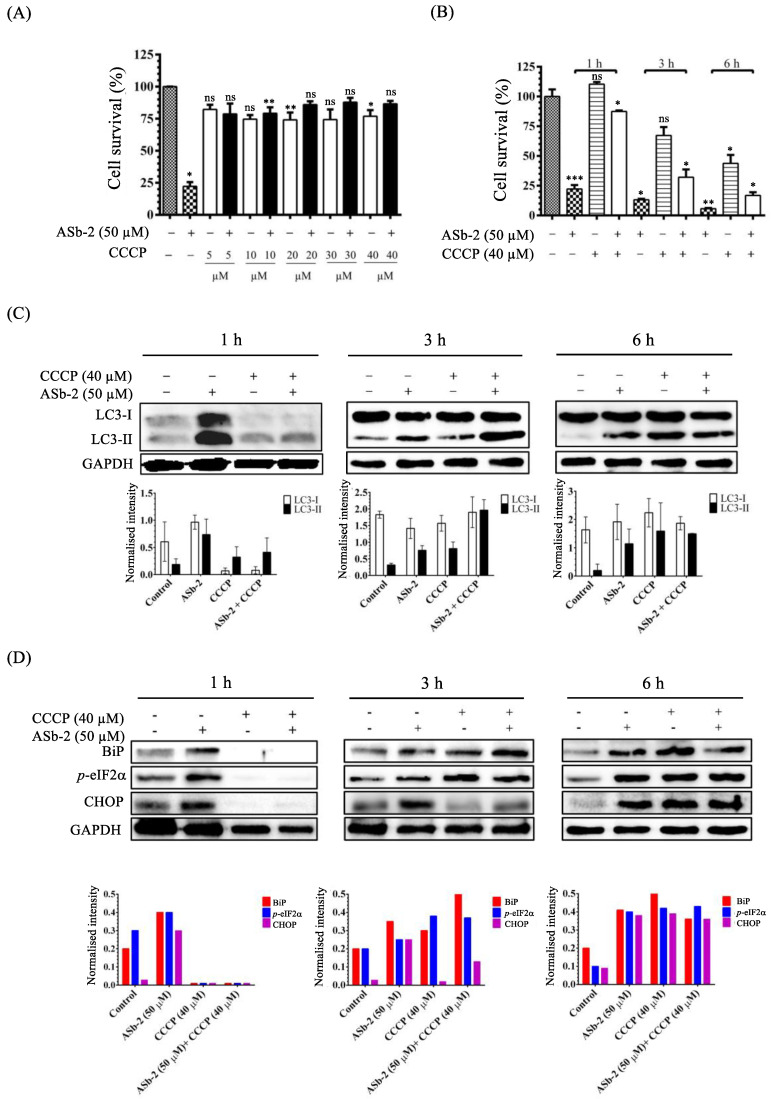
(**A**) The results of MTT assays of Jurkat cells treated with ASb-2 (50 µM) in the presence of CCCP (0–40 µM) for 1 h at 37 °C. (**B**) The results of MTT assay of Jurkat cells that had been treated with ASb-2 in the presence of CCCP (40 µM) for 1, 3, and 6 h at 37 °C. (**C**) Western blot analysis of LC3-I/II expression of Jurkat cells that had been treated with ASb-2 (50 µM) in the presence of CCCP (40 µM) for 1, 3, and 6 h at 37 °C. (**D**) Western blot analysis of BiP, *p*-eIF2α and CHOP in Jurkat cells treated with ASb-2 in the presence of CCCP for 1, 3, and 6 h at 37 °C. * *p* < 0.05, ** *p* < 0.01, and *** *p* < 0.001 indicate the significant differences from the control. ns: no significant (the statistical data in Figure 14A,B are listed in Table S5 in the Supporting Information).

**Figure 15:**
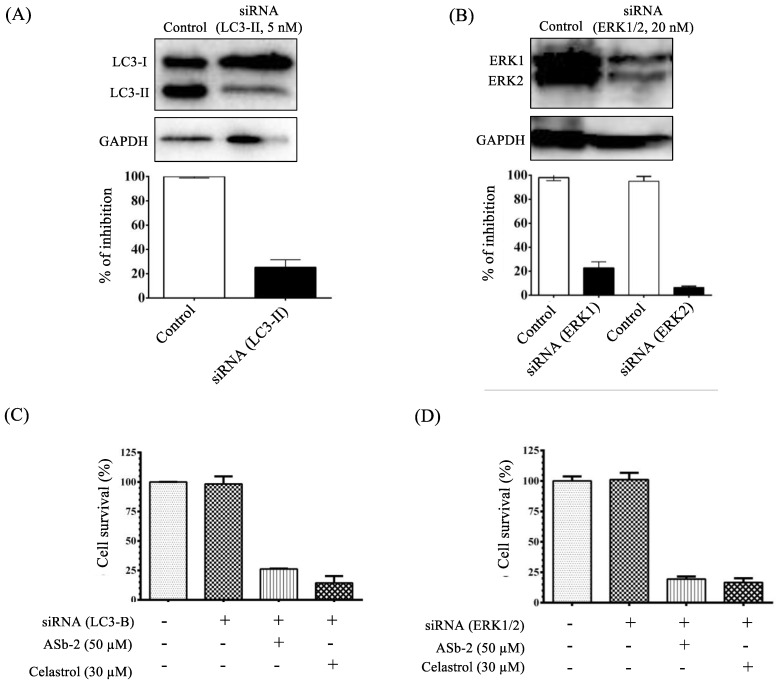
(**A**) Western blot results of LC3-II knock-down by siRNA-LC3-II (5 nM) in Jurkat cells. (**B**) Western blot results of ERK1/2 knock-down by siRNA-ERK1/2 (20 nM) in Jurkat cells. (**C**) The results of MTT assays of Jurkat cells treated with siRNA (LC3-II), ASb-2, and celastrol. (**D**) The results of MTT assays of Jurkat cells treated with siRNA (ERK1/2), ASb-2, and celastrol.

The relationship between ASb-2-induced paraptosis and ER stress was examined by using CCCP, a compound that inhibits the paraptosis induced by ASb-2. As shown in Figure 14A, the pretreatment of Jurkat cells with CCCP (5–40 µM) inhibits the cell death induced by ASb-2 to a considerable extent, thus confirming the results shown in Figure 5 and Figure 13 (Figure 14B implies that the inhibitory effect of CCCP is reduced after incubation for 3 and 6 h, possibly due to the slow toxicity of CCCP itself). The results of Western blot analyses of LC3-I/II after the treatment of Jurkat cells with CCCP (40 µM) for 1 h and then with ASb-2 (50 µM) for 1~6 h show that LC3-I/II expression is inhibited by CCCP to a considerable extent after incubation for 1 h, while the expression of LC3-II is upregulated after incubation for 3 and 6 h (Figure 14C). As shown in Figure 14D, similar phenomena were observed for ER stress markers such as BiP, *p*-eIF2α, and CHOP. Western blot results suggest that CCCP alone and combination of CCCP and ASb-2 downregulate BiP expression after the treatment for 1 h. Besides, the re-expression of BiP protein was observed after the incubation for 3 and 6 h with CCCP and CCCP + ASb-2. We carried out the knock-down of LC3-II and ERK1/2 by using the corresponding small interfering RNA (siRNA) (76% inhibition of LC3-II, 78% inhibition of ERK-1, and 95% inhibition of ERK-2, as shown in Figure 15A,B and Figure S1 in the Supporting Information). These knockdowns were found to negligibly affect the cell death of Jurkat cells induced by ASb-2 (50 µM) and celastrol (30 µM), as shown in Figure 15C,D. The collective data indicate that the expression and/or the upregulation of LC3-I/II, BiP, CHOP, and so on, by ASb-2 is possibly the result of ASb-2-induced paraptosis and not the cause of this type of PCD. It should be mentioned that the changes in the expression levels of LC3-I/II and CHOP are rapid and it is unlikely that these proteins are produced with relatively slow protein neosynthesis. We assume the possibility that these marker proteins or their precursors already exist before treatment with ASb-2 and then undergo processing and/or structural changes to their active forms after stimulation with ASb-2.

## 3. Discussion

**Scheme 1:**
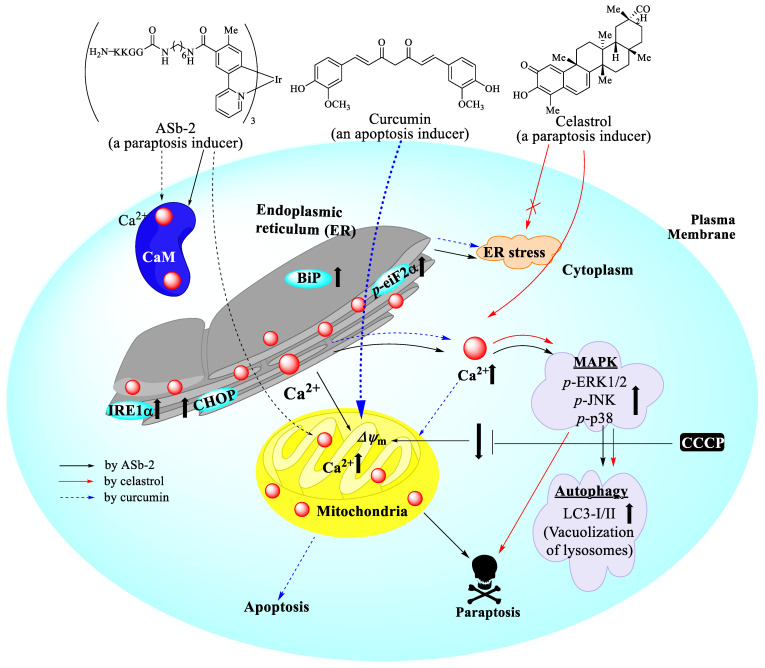
The proposed mechanism for paraptosis-type programmed cell death induced by ASb-2 (black plain arrows), celastrol (red plain arrows), and curcumin (blue dashed arrows) in Jurkat cells.

The results of mechanistic studies concerning cell death in Jurkat cells induced by ASb-2 with typical examples of IPHs (celastrol, hesperidin, and curcumin) (Figure 1) are summarized below and in Scheme 1.

1. ASb-2 and celastrol induce paraptotic cell death in Jurkat cells rather than apoptosis and necroptosis-mediated cell death, as confirmed by TEM analysis, confocal microscopic observations using Z-VAD-FMK and necrostatin-1, and Western blot analyses (Figure 4).
2. ASb-2 induces the transfer of Ca^2+^ possibly from the ER to mitochondria in 30 min (Figure 6A–F) and reduces the mitochondrial membrane potential (Δ*Ψ_m_*) (Figure 10A) to induce paraptosis, while celastrol increases the cytoplasmic Ca^2+^ concentration in 2–4 h (Figure 6G–L) and negligibly alters the Δ*Ψ_m_* (Figure 10). Curcumin, an apoptosis inducer, promotes Ca^2+^ overload in mitochondria and cytoplasm (Figure 9), while it induces a small increase in Δ*Ψ_m_* (Figure 10D). The direct transport of Ca^2+^ from the ER to mitochondria (not from outside the cell) is supported by the experimental finding that the cell death induced by ASb-2 was not inhibited by BAPTA-AM, a cytosolic Ca^2+^ chelator (Figure 5A,B), and was not affected by the use of a Ca^2+^-free medium (Figure 8). It has been reported that a close contact exists between the ER and mitochondria and that the Ca^2+^ transport systems between them play important roles, not only in cell survival but also in cell death [67,68,69,70,71]. It is also well known that an overload of intracellular Ca^2+^ or a perturbation in intracellular Ca^2+^ compartmentalization induces cell death (apoptosis, necroptosis, paraptosis, or autophagy) either in caspase-dependent or -independent manners [72,73,74,75,76].
3. ASb-2 induces the degradation of lysosomes and the ER (Figure 11A and Figure 13A). In contrast, celastrol induces the degradation of lysosomes (Figure 11B) but not the degradation of the ER (Figure 13B).
4. Although the expression of LC3-I/II, a typical autophagy marker, was dramatically changed after the treatment of Jurkat cells with ASb-2 and celastrol (Figure 11C,D), negligible inhibition of cell death was observed in the presence of 3-MA and CQ (Figure 11F–J), suggesting that autophagy is not responsible for the paraptosis caused by ASb-2 and celastrol. Moreover, the phosphorylation of ERK, JNK, and p38, members of the MAPK signaling pathway, was gradually upregulated by ASb-2 and celastrol and this upregulation was dose-dependent (Figure 12A,B). However, the pre-treatment with MAPK inhibitors such as SCH772984, SP600125, U0126, and SB203580 had little effect on blocking the cell death by ASb-2 (1 h) and celastrol (24 h) (Figure 12C–H). These results suggest that the MAPK signaling pathway is not the main pathway for ASb-2-induced paraptosis.
5. Based on the aforementioned results, we speculate that cell death possibly involves the inhibition of the complexation of Ca^2+^ with CaM or the interaction of the Ca^2+^–CaM complex with its downstream target proteins [44], followed by an extensive overload of Ca^2+^ from the ER to mitochondria (Figure 6A–F), which is associated with ER stress (Figure 13A,C). These phenomena induce the loss of Δ*Ψ_m_* (Figure 10A–D) related to mitochondrial dysfunction, resulting in a paraptosis with vacuolization of intracellular organelles such as lysosomes and the ER in Jurkat cells (Scheme 1). On the other hand, ER stress markers had a negligible effect after treatment with celastrol (Figure 13D), which increases cytoplasmic Ca^2+^ concentrations to induce paraptosis in Jurkat cells, suggesting that ASb-2 and celastrol induce paraptosis via different signaling pathways. It has been reported that the ER and mitochondria are main reservoirs for intracellular Ca^2+^ [73] and that damage to the ER and mitochondria by Ca^2+^ distribution, Ca^2+^ overload, and a loss of Ca^2+^ homeostasis induces cytotoxicity, oxidative stress, and mitochondrial dysfunction, resulting in PCD such as paraptosis [77], although the molecular target of ASb-2 has not been precisely identified. It was also found that curcumin increases cytoplasmic and mitochondrial Ca^2+^ concentrations and ER stress to induce apoptosis in Jurkat cells (Figure 9C,D and Figure 13E,F).

## 4. Materials and Methods

### 4.1. Chemicals and Reagents

3-(4,5-Dimethyl-2-thiazolyl)-2,5-diphenyl-2H-tetrazolium bromide (MTT), Rhod-2/AM, and Rhod-4/AM were purchased from Dojindo (Kumamoto, Japan). Z-VAD-fmk was purchased from the Peptide Institute and necrostatin-1 was purchased from Enzo Life Science. Opti-MEM media was purchased from Gibco (Grand Island, USA) and acridine orange was purchased from Nacalai Tesque (Kyoto, Japan). Ethidium bromide (EB), ethylene glycol-bis(*β*-aminoethylether)-*N,N,N*′*,N*′-tetraacetic acid (EGTA), the radioimmunoprecipitation assay (RIPA) buffer, Blocking One solution, HIKARI-solution, ChemiLumi One Ultra solution, and NaN_3_ were purchased from Nacalai Tesque, Japan, and 3-methyladenine, chloroquine diphosphate, bovine serum albumin (BSA), benzylpenicillin potassium, streptomycin sulfate, and monothioglycerol (MTG) were purchased from WAKO Pure Chemical Industries (Osaka, Japan). The Lipofectamine RNAiMAX reagent, ER-RFP, ER-Tracker Red, and LysoTracker Red DND-99 were purchased from Invitrogen and small interfering RNA (siRNA) (LC3-II and ERK1/2), carbonyl cyanide 3-chlorophenylhydrazone (CCCP), and 1,1′,3,3,3′,3′-hexamethylindodicarbocyanine iodide (DilC1(5)) were purchased from Sigma Aldrich (Tokyo, Japan). Rapamycin, cycloheximide, antibodies against caspase-3, PARP, Akt, *p*-Akt, ERK1/2, *p*-p38, and *p*-ERK1/2 were purchased from Santa Cruz Biotechnology (Dallas, TX, USA). Antibodies against BiP, IRE1α, eIF2α, *p*-eIF2α, JNK1/2, *p*-JNK1/2, CHOP, LC3-I/II, Atg12, Beclin-1, mTOR, GAPDH, and horse-radish peroxidase (HRP)-conjugated anti-rabbit and anti-mouse secondary antibodies were purchased from Cell Signaling Technology (Beverly, MA, USA). Celastrol, U0126, 2-aminoethoxydiphenyl borate (2-APB), SP600125, SCH772984, SB203580, and tauroursodeoxycholic acid (TUDCA) were purchased from Cayman Chemical Co. (Ann Arbor, MI, USA). Curcumin, hesperidin, and 1,2-bis(2-aminophenoxy)ethane-*N*,*N*,*N*′,*N*′-tetraacetic acid tetrakis(acetoxymethyl ester) (BAPTA-AM) were purchased from Tokyo Chemical Industry Co., Ltd. (TCI) (Tokyo, Japan), and hesperidin was purified by recrystallization from MeOH/H_2_O (1/1) because the purity of commercially available hesperidin was ca. 90%.

### 4.2. MTT Assay

*(a) Cytotoxic study:* Jurkat, K562 and U937 cells were cultured and incubated in RPMI 1640 medium containing 10% (*v*/*v*) fetal bovine serum (FBS) and 1% (*v*/*v*) penicillin/streptomycin under a 5% CO_2_ atmosphere at 37 °C. A549, HeLaS3 and IMR-90 cells were cultured and incubated in DMEM containing 10% (*v*/*v*) fetal bovine serum (FBS) and 1% (*v*/*v*) penicillin/streptomycin under a 5% CO_2_ atmosphere at 37 °C. Jurkat cells (2 × 10^4^ cells/well) were seeded in 96-well plates and treated with ASb-2 (IPH) (100 to 0.05 µM) (1, 3, 6, 12, and 24 h), celastrol (100 to 0.05 µM) (1, 3, 6, 12, and 24 h), curcumin (100 to 0.05 µM) (24 h), or hesperidin (100 to 16 µM) (24 h) under a 5% CO_2_ atmosphere at 37 °C for a given incubation time. The cells were then incubated with a solution of MTT (5 mg/mL) in PBS (10 µL) at 37 °C for 4 h, to which a 10% SDS in 0.01 N HCl aqueous solution (100 µL) was added to dissolve formazan (a reduced product of MTT), and the resulting solution was incubated overnight under the same conditions. The results of the MTT assay were measured by a microplate reader (Bio-Rad, Hercules, CA, USA) at the absorbance of 570 nm. Vehicle controls were used in the experiment.

*(b) MTT assays in the presence of various inhibitors:* Jurkat cells (2 × 10^4^ cells/well) were pretreated with Z-VAD-fmk (a broad caspase inhibitor) (15 µM), necrostatin-1 (a necroptosis inhibitor) (30 µM), chloroquine (CQ) (25 µM) and 3-methyladenine (3-MA) (5 mM) (autophagy inhibitors), SB203580 (a p38 inhibitor) (20 µM), U0126 (a MEK1 inhibitor) (10 µM), SCH772984 (an ERK1/2 inhibitor) (1 µM), SP600125 (a JNK inhibitor) (20 µM), CCCP (carbonyl cyanide 3-chlorophenylhydrazone) (a mitochondrial uncoupling reagent) (40 µM), rapamycin (a mTOR inhibitor) (200 nM), cycloheximide (an inhibitor of protein synthesis) (2 µM), 2-APB (2-aminoethoxydiphenyl borate) (an inhibitor of IP₃ receptors) (50 µM), BAPTA-AM (a cytosolic Ca^2+^ chelator) (10 µM), TUDCA (tauroursodeoxycholic acid) (an enhancer of protein folding and protector of cells against ER stress) (40 µM), or EGTA (ethylene glycol-bis(*β*-aminoethylether)-*N,N,N*′,*N*′-tetraacetic acid) (a Ca^2+^ chelator) (0.5, 1, or 2 mM) for 1 h, followed by the treatment with ASb-2 (50 µM) under a 5% CO_2_ atmosphere at 37 °C for 1 h. To test the effect of celastrol, the cells were pretreated with the above-mentioned inhibitors of autophagy and MAPK under a 5% CO_2_ atmosphere at 37 °C for 1 h and then incubated with celastrol (1 µM) for 24 h. To check the effect of CCCP on the cytotoxicity of ASb-2 in a time- and concentration-dependent manner, Jurkat cells were pre-treated with CCCP (5 to 40 µM or 40 µM) for 1 h or 1–6 h, followed by the treatment with ASb-2 (50 µM) under a 5% CO_2_ atmosphere at 37 °C. The results of the MTT assays were obtained as described above. Vehicle controls were used in the experiment.

*(c)The Trypan Blue dye exclusion test:* Jurkat cells were cultured and incubated in RPMI 1640 medium containing 10% (*v*/*v*) fetal bovine serum (FBS) and 1% (*v*/*v*) penicillin/streptomycin under a 5% CO_2_ atmosphere at 37 °C. Jurkat cells (2 × 10^4^ cells/well) were seeded in 96-well plates and treated with ASb-2 (IPH) and celastrol (50 to 0.04 µM) under a 5% CO_2_ atmosphere at 37 °C for 24 h. After the treatment cells were mixed with Trypan blue (0.4%) solution 1:1 ratio and cells were counted. Vehicle controls were used in the experiment.

### 4.3. Fluorescent Microscopic Studies of Jurkat Cells in the Presence of Various Inhibitors

Jurkat cells (1.0 × 10^6^ cells/mL, 100 μL) were pre-treated with CQ (25 µM), 3-MA (5 mM), SB203580 (20 µM), U0126 (10 µM), SCH772984 (1 µM), SP600125 (20 µM), CCCP (40 µM), rapamycin (200 nM), cycloheximide (2 µM), 2-APB (50 µM), BAPTA-AM (10 µM), or TUDCA (40 µM) in RPMI 1640 medium containing 10% (*v*/*v*) FBS and 1% (*v*/*v*) penicillin/streptomycin for 1 h and followed by the treatment with ASb-2 (50 µM) under a 5% CO_2_ atmosphere at 37 °C for 1 h. After the treatment, the cells were washed twice with PBS and images were then observed by fluorescent microscopy (Biorevo, BZ-9000, Keyence, Osaka, Japan) (excitation at 377 nm, emission at 520 nm).

### 4.4. Confocal Microscopic Images of Jurkat Cells

Jurkat cells (1.0 × 10^6^ cells/mL, 100 μL) were maintained in RPMI 1640 medium containing 10% (*v*/*v*) FBS and 1% (*v*/*v*) penicillin/streptomycin under a 5% CO_2_ atmosphere at 37 °C. Vehicle controls were used in the experiment.

*(a) Observation of alive and dead cells:* Jurkat cells were treated with ASb-2 (50 µM) for 1 h and celastrol (1 µM) for 24 h. After the treatment, the cells were washed with twice PBS and incubated in an ethidium bromide solution (EB, 100 µM) for 15 min in the dark. For celastrol, cells were washed twice with PBS and incubated with AO (200 µM) and ethidium bromide (EB, 100 µM) for 15 min in the dark. The cells were washed with PBS and then observed by confocal microscopy (Fluoview, FV-1000, Olympus, Tokyo, Japan) using a Greiner CELLview^TM^ Petri dish (35 × 10 mm). Excitation at 405 nm and emissions from 520 to 560 nm were used for ASb-2. Excitation at 473 nm and emissions at 485–545 nm were used for AO. Excitation at 559 nm and emissions at 570–620 were used for EB.

*(b) Measurement of O_2_ level:* Jurkat cells were treated with ASb-2 (50 µM) for 1 h, after which the cells were washed twice with PBS and incubated BTPDM1 (3 µM) [47,48,49] at 37 °C for 3 h. The cells were washed with PBS and then observed by confocal microscopy as described above. Excitation at 473 nm and emissions at 680–730 nm were used for BTPDM1.

*(c) Measurement of intracellular Ca^2+^ level:* Jurkat cells were treated with Rhod-4/AM (5 µM) or Rhod-2/AM (5 µM) for 30 min, after which the cells were washed twice with PBS and incubated with ASb-2 (50 µM), celastrol (30 µM), or curcumin (50 µM) for 0–4 h. After the incubation, the cells were washed with PBS and then observed by confocal microscopy as described above. Excitation at 559 nm and emissions from 570 to 620 nm were used for Rhod-2 and Rhod-4.

*(d) Observation of lysosome:* Jurkat cells were treated with ASb-2 (50 µM) or celastrol (30 µM) for 1 or 24 h, after which the cells were washed twice with PBS and incubated with LysoTracker Red (100 nM) under a 5% CO_2_ atmosphere at 37 °C for 1 h. After the incubation, the cells were washed with PBS and then observed by confocal microscopy as described above. Excitation at 559 nm and emissions at 610–680 nm were used for LysoTracker Red.

*(e) Observation of endoplasmic reticulum (ER):* Jurkat cells were treated with ASb-2 (50 µM) and curcumin (50 µM) for 1 h, and for celastrol, Jurkat cells were treated with 30 µM for 1 or 24 h. After the treatment cells were washed with PBS and collected by centrifugation, the cells were stained with ER-Tracker Red (1 µM) under a 5% CO_2_ atmosphere at 37 °C for 1 h. After the incubation, cells were washed with PBS and images were observed on confocal microscopy as described above. Excitation at 559 nm and emissions at 610–680 nm were used for ER-Tracker Red.

*(f) Measurement of mitochondrial membrane potential (*Δ*Ψ_m_):* Jurkat cells were treated with ASb-2 (50 µM), celastrol (30 µM), or curcumin (50 µM) for 1 h. After the treatment, the cells were washed twice with PBS and incubated with DilC1(5) (500 nM) under a 5% CO_2_ atmosphere at 37 °C for 30 min. After the incubation, the cells were washed with PBS and then observed by confocal microscopy as described above. Excitation at 635 nm and emissions at 650–700 nm were used for DilC1(5).

### 4.5. Transmission Electron Microscopy (TEM) Analysis of Jurkat Cells Treated with ASb-2 and Cleastrol

Jurkat cells (3 × 10^6^ cells) were treated with ASb-2 (50 µM, 1 h) or celastrol (1 µM, 24 h) in RPMI 1640 medium containing 10% (*v*/*v*) FBS and 1% (*v*/*v*) penicillin/streptomycin under a 5% CO_2_ atmosphere at 37 °C. After the treatment, the cells were washed with ice-cold PBS and then prefixed by treatment with glutaraldehyde (2.5%) at 4 °C for 40 min. After the prefixation, the cells were washed with ice-cold PBS and post-fixation was conducted with osmium tetroxide (1%) at 4 °C for 30 min. The cells were then washed and included in an agarose gel, and dehydrated by treatment with 50–100% and anhydrous EtOH. Embedding of the cells in Poly 812 resin (Nisshin EM Co. Ltd., Tokyo, Japan) was conducted at 60 °C for 3 days. The resin was sliced by a glass knife (100 nm thickness) on an ultramicrotome (EM UC6, Leica). The sliced samples (ca. 100 nm thickness) were stained (EM stainer, Nisshin EM Co. Ltd.) and images were obtained by a TEM instrument (H-7650, HITACHI) with electron irradiation at 100 kV. Vehicle controls were used in the experiment.

### 4.6. Flow Cytometric Analysis

*(a) Measurement of intracellular Ca^2+^ level:* Jurkat cells (1.0 × 10^6^ cells/mL, 100 μL) were treated with Rhod-4/AM (5 µM) or Rhod-2/AM (5 µM) in RPMI 1640 medium containing 10% (*v*/*v*) FBS and 1% (*v*/*v*) penicillin/streptomycin under a 5% CO_2_ atmosphere at 37 °C for 30 min. After staining, the cells were washed twice with PBS and incubated with ASb-2 (50 µM), celastrol (30 µM), or curcumin (50 µM) under a 5% CO_2_ atmosphere at 37 °C for a given incubation time. After the incubation, the cells were washed with PBS and analyzed by flow cytometry (FACS Calibur cytometer, Becton), and the data were analyzed on FlowJo software (FlowJo, LCC). Vehicle contols were used in the experiment.

*(b) Measurement of mitochondrial membrane potential (ΔΨ_m_):* Jurkat cells (1.0 × 10^6^ cells/mL, 100 μL) were treated with ASb-2 (50 µM), celastrol (30 µM), or curcumin (50 µM) in RPMI 1640 medium under a 5% CO_2_ atmosphere at 37 °C for 1 h. After the treatment, the cells were washed twice with PBS and incubated with DilC1(5) (500 nM) for 30 min under a 5% CO_2_ atmosphere at 37 °C. After the incubation, the cells were washed with PBS and analyzed by flow cytometry as described above. Vehicle controls were used in the experiment.

### 4.7. Western Blot Analysis

(a) Jurkat cells (3 × 10^6^ cells/mL) were treated with ASb-2 (10 to 50 µM), celastrol (30 to 50 µM), or curcumin (25 to 100 µM) under a 5% CO_2_ atmosphere at 37 °C for 1 h and cisplatin (25 to 50 µM) for 24 h.

(b) For autophagy and MAPK inhibitors, Jurkat cells were pre-treated with Z-VAD-fmk (15 µM), CQ (25 µM), 3-MA (5 mM), SB203580 (20 µM), U0126 (10 µM), SCH772984 (1 µM), or SP600125 (20 µM) for 1 h, followed by the treatment with ASb-2 (50 µM) under a 5% CO_2_ atmosphere at 37 °C for 1 h.

(c) Jurkat cells were incubated in the presence or absence of CCCP (40 µM) for 1 h, followed by the treatment with ASb-2 (50 µM) for 1 h, 3 h, and 6 h.

After the treatment (a, b, and c), the cells were washed twice with ice-cold PBS, incubated with RIPA buffer (Nacalai Tesque, Kyoto, Japan) for 5 min, and vortexed for 1 min. The cells were then incubated at 4 °C for 15 min and cell mixture was centrifuged at 10,000 rpm at 4 °C for 15 min. After centrifugation, the lysate was transferred to a new 1.5 mL tube and the extracted proteins were quantified using the Pierce^TM^ BCA Protein Assay Kit (Thermo Scientific). Proteins (50 µg/well) were used for SDS-PAGE. After SDS-PAGE, the gel was transferred to polyvinylidene fluoride membranes using a semi dry blotter (Bio-Rad) and the membrane was blocked with Blocking One solution (Nacalai Tesque, Japan) at room temperature for 30 min. After blocking, the membrane was washed 3 times with 1’TBST (5 min at each time) and incubated overnight with primary antibodies (caspase-3, PARP, Akt, *p*-Akt, ERK1/2, *p*-ERK1/2, BiP, IRE1α, eIF2α*,* *p*-eIF2α, JNK1/2, *p*-JNK1/2, *p*-p38, CHOP, LC3-I/II, Atg12, Beclin-1, mTOR, and GAPDH) that were diluted with HIKARI-solution A (Nacalai Tesque, Japan) (a dilution of 1:1000). The membrane was washed 3 times with TBST and incubated with an HRP-conjugated anti-rabbit or anti-mouse secondary antibody (a dilution of 1:5000) diluted with signal enhancer HIKARI-solution B (Nacalai Tesque, Japan) at room temperature for 60 min. The protein signal was spotted by Chemi-Lumi One Ultra solution (Nacalai Tesque, Japan) using ChemiDoc MP system (Bio-Rad, California, USA). Vehicle controls were used in the experiment.

### 4.8. Knockdown of the LC3-II and ERK 1/2 Genes by siRNA (Small Interfering RNA)

Jurkat cells (2.5 × 10^5^ cells/mL) were seeded in 12-well plates in Opti-MEM (1x) medium (500 µL) containing Lipofectamine RNAiMAX (5 µL) and LC3-II (5′-GAGAUCUCCCUAAGAGGAU-3′ and 5′-AUCCUCUUAGGGAGAUCUC-3′) siRNA (5 nM) or ERK1/2 (5′-GACACAACACCUCAGCAAU-3′ and 5′-AUUGGUGAGGUGUUGUGUC-3′) siRNA (20 nM) under a 5% CO_2_ atmosphere at 37 °C for 47 h. After the treatment, the cells were treated with ASb-2 (50 µM) or celastrol (30 µM) under a 5% CO_2_ atmosphere at 37 °C for 1 h. MTT solution (5 mg/mL) in PBS (10 µL) was added and incubated at 37 °C for 4 h and then 10% SDS in 0.01 N HCl aqueous solution (100 µL) was added, followed by incubation overnight under the same conditions. The results of the MTT assays were measured by a microplate reader (Bio-Rad, CA, USA) at the absorbance of 570 nm. The knockdown experiment on LC3-II and ERK1/2 using siRNA was confirmed by the above-mentioned Western blot method.

### 4.9. Statistical Analysis

All the experiments were carried out in triplicate and the data were calculated by SD ± mean using Graphpad Prism 6 software. Statistical analyses were performed with one-way ANOVA and the significance was calculated by Tukey’s multiple comparisons test (*p* < 0.05).

## 5. Conclusions

In summary, we report on the mechanism of PCD in Jurkat cells induced by ASb-2, a tris-cyclometalated Ir(III) complex containing cationic KKGG peptide units (which has dual functions as an inducer of paraptosis in Jurkat cells and a green-emission probe of dead cells), as compared with celastrol, a naturally-occurring anticancer agent. Detailed mechanistic studies indicate that ASb-2 enhances mitochondrial Ca^2+^ concentrations, possibly via direct transfer of Ca^2+^ from the ER, resulting in a decrease in Δ*Ψ_m_*, the induction of ER stress, and the induction of cytoplasmic vacuolation in Jurkat cells, which are characteristic phenomena of paraptosis. Although the upregulation of autophagy-related and mitogen-activated protein kinase (MAPK)-related proteins was observed in Jurkat cells treated with ASb-2, the treatment of Jurkat cells with the inhibitors of autophagy and MAPK signaling pathways prior to the treatment with ASb-2 had little effect on cell death, indicating that autophagy and the MAPK signaling pathway are not the main causes of ASb-2-induced paraptosis. On the other hand, celastrol enhances the cytoplasmic Ca^2+^ concentrations that induce paraptosis by MAPK and the autophagy signaling pathway. Therefore, we conclude that ASb-2 and celastrol induce paraptosis in Jurkat cells via different intracellular signaling pathways (possibly not only in Jurkat cells but also in other cancer cells). The findings reported in this work suggest the possibility that an ER–mitochondria tethering site could be a target for cancer chemotherapy, and a more detailed study is now underway. In addition, we conclude that the development of organometallic compounds such as IPHs (which hopefully have more potent anticancer activity) will afford a new approach for revealing biological phenomena and preventing or fighting malignant cells, tumors, and related diseases.

## Data Availability

The data presented in this study are available in article.

## Supplementary Materials

The following are available online, Figure S1: Knockdown of LC3-II and ERK1/2 by siRNA (Western blot analysis), Table S1: Statistical data of Figure 4A,B, Table S2: Statistical data of Figure 5A,C, Table S3: Statistical data of Figure 11F,H, Table S4: Statistical data of Figure 12F,G and Table S5: Statistical data of Figure 14A,B.

## Sample Availability

Samples of the compound ASb-2 is available from the authors.
