# Supplementary material for: Cyclometalated Iridium(III) Complex–Cationic Peptide Hybrids Trigger Paraptosis in Cancer Cells via an Intracellular Ca2+ Overload from the Endoplasmic Reticulum and a Decrease in Mitochondrial Membrane Potential
Source: Molecules. 2021 Nov 21;26(22):7028. doi: 10.3390/molecules26227028 (PMC8623854; doi:10.3390/molecules26227028)
Supplement: Supplementary file 1 [file molecules-26-07028-s001.zip › molecules-1310913-supplementary.pdf]

Cyclometalated Iridium(III) Complex-Cationic Peptide Hybrids Trigger Paraptosis in Cancer Cells via an Intracellular Ca^{2+} Overload from the Endoplasmic Reticulum and a Decrease in Mitochondrial Membrane Potential

Chandrasekar Balachandran,[1,2] Kenta Yokoi,[1] Kana Naito,[1] Jebiti Haribabu,[1] Yuichi Tamura,[1] Masakazu Umezawa,[3] Koji Tsuchiya,[3] Toshitada Yoshihara,[4] Seiji Tobita,[4] and Shin Aoki*[1,2,3]

[1] Faculty of Pharmaceutical Sciences, Tokyo University of Science, 2641 Yamazaki, Noda, Chiba 278-8510, Japan

[2] Research Institute for Biomedical Sciences (RIBS), Tokyo University of Science, 2641 Yamazaki, Noda, Chiba 278-8510, Japan

[3] Research Institute for Science and Technology (RIST), Tokyo University of Science, 2641 Yamazaki, Noda, Chiba 278-8510, Japan

[4] Department of Chemistry and Chemical Biology, Graduate School of Science and Technology, Gunma University, 1-5-1 Tenjin-cho, Kiryu-shi, Gunma 376-8515, Japan

* Corresponding author: E-mail, shinaoki@rs.tus.ac.jp; Tel.:+81-4-7121-3670

Contents

1. **Table S1.** Statistical data of Figure 4A and B 2

2. **Table S2.** Statistical data of Figure 5A and C.. 3

3. **Table S3**. Statistical data of Figure 11F and H.. 4

4. **Table S4.** Statistical data of Figure 12F and G 5

5. **Table S5.** Statistical data of Figure 14A and B 6

6. **Figure S1.** Knockdown of LC3-II and ERK1/2 by siRNA (Western blot analysis)................ 7

Table S1. Statistical data of Figure 4A and B in the text.

Apoptosis

	Control	ASb-2 (50 µM)	Necrostatin (30 µM)	Necrostatin + ASb-2
	99.063	25.117	102.576	31.792
	102.049	20.023	105.738	27.752
	98.888	13.349	113.466	21.780
Mean	100.000	19.496	107.260	27.108
SD	1.777	5.902	5.602	5.037

Necrosis

	Control	ASb-2 (50 µM)	Z-VAD-FMK (15 µM)	Z-VAD-FMK + ASb-2
	105.246	23.213	112.328	22.033
	102.885	18.885	113.705	18.885
	91.869	16.721	97.771	14.557
Mean	100.000	19.607	107.934	18.492
SD	7.140	3.305	8.829	3.753

Table S2. Statistical data of Figure 5A and C in the text.

ASb-2

	control	ASb-2 (50 μM)	CCCP (40 μM)	CCCP + ASb-2	Rapamycin (200 nM)	Rapamycin + ASb-2	Cycloheximide (2 μM)	Cycloheximide + ASb-2
	100.962	18.214	112.549	81.335	97.303	18.367	99.486	25.312
	99.462	20.760	111.220	88.146	106.050	19.461	110.051	24.431
	98.450	15.288	109.339	93.889	105.175	20.335	109.391	25.532
Mean	99.625	18.088	111.036	87.790	102.843	19.388	106.310	25.092
SD	1.264	2.738	1.613	87.790	4.817	0.986	5.918	0.582

2-APB (10 μM)	2-APB + ASb-2	BAPTA-AM (10 μM)	BAPTA-AM + ASb-2	TUDCA (40 μM)	TUDCA + ASb2
63.300	52.445	68.480	23.857	97.167	36.317
55.785	44.720	57.856	19.124	92.144	35.834
55.993	38.875	65.390	20.766	92.820	32.164
58.359	45.347	63.909	21.249	94.044	34.771
4.280	6.806	5.465	2.403	2.726	2.271

Celastrol

	control	Celasrtol (1 μM)	CCCP (40 μM)	CCCP + celastrol	Rapamycin (200 nM)	Rapamycin + celastrol	Cycloheximide (2 μM)	Cycloheximide + celastrol
	99.336	2.187	6.410	4.034	70.669	7.396	38.057	4.924
	101.389	1.065	9.112	4.133	74.914	7.890	43.074	4.825
	100.258	1.187	9.691	4.528	86.106	9.373	39.022	7.297
Mean	100.328	1.479	8.404	4.232	77.230	8.220	40.051	5.682
SD	1.028	0.615	1.751	0.262	7.975	1.029	2.662	1.399

2-APB (10 μM)	2-APB + celastrol	BAPTA-AM (10 μM)	BAPTA-AM + celastrol	TUDCA (40 μM)	TUDCA + celastrol
20.304	4.726	9.318	4.881	88.245	8.682
20.690	6.011	12.208	9.705	94.236	8.719
17.796	6.802	13.321	6.321	84.378	10.468
19.597	5.847	11.616	6.969	88.953	9.290
1.572	1.048	2.066	2.477	4.967	1.020

Table S3. Statistical data of Figure 11F and H in the text.

ASb-2

	Control	ASb-2 (50 µM)	CQ (25 µM)	CQ + ASb-2	3-MA (5 mM)	3-MA + ASb-2
	99.044	26.573	100.965	18.428	73.996	16.980
	96.078	26.211	101.146	20.057	71.281	16.075
	97.129	26.030	105.490	20.057	75.263	16.075
mean	97.417	26.271	102.534	19.514	73.513	16.377
SD	1.504	0.276	2.562	0.941	2.034	0.523

Celastrol

	Control	Celastrol (1 µM)	CQ (25 µM)	CQ + celastrol	3-MA (5 mM)	3-MA + celastrol
	100.682	1.587	98.263	7.396	55.038	6.605
	98.719	0.499	78.195	8.187	63.336	8.384
	102.468	0.646	67.774	8.582	54.267	8.582
Mean	100.623	0.910	81.411	8.055	57.547	7.857
SD	1.875	0.590	15.497	0.604	5.028	1.089

Table S4. Statistical data of Figure 12F and G in the text.

ASb-2

	Control	ASb-2 (50 μM)	SCH772984 (1 μM)	SCH772984 + ASb-2	SP600125 (20 μM)	SP600125 + ASb-2
	99.766	22.834	89.930	21.956	64.930	25.312
	97.658	24.415	95.550	23.185	66.031	25.312
	102.576	28.103	99.766	26.347	59.868	24.872
Mean	100.000	25.117	95.082	23.829	63.610	25.165
SD	2.467	2.704	4.935	2.265	3.287	0.254

U0126 (10 μM)	U0126 + ASb-2	SB203580 (20 μM)	SB203580 + ASb-2
108.740	48.478	95.658	22.801
98.814	50.148	91.274	80.210
117.572	50.148	94.405	24.262
108.375	49.592	93.779	42.424
9.384	0.964	2.258	32.731

Celastrol

	Control	Celastrol (1 μM)	SCH772984 (1 μM)	SCH772984 + celastrol	SP600125 (20 μM)	SP600125 + celastrol
	98.070	0.597	102.123	11.054	12.006	6.506
	102.316	0.756	106.368	10.065	12.199	7.099
	99.614	1.054	115.437	11.647	14.901	8.285
Mean	100.000	0.802	107.976	10.922	13.036	7.297
SD	2.149	0.232	6.802	0.799	1.618	0.906

U0126 (10 μM)	U0126 + celastrol	SB203580 (20 μM)	SB203580 + celastrol
98.701	9.472	74.721	15.287
107.910	9.373	71.634	23.392
114.348	10.658	65.073	20.497
106.986	9.834	70.476	19.725
7.865	0.715	4.927	4.107

Table S5. Statistical data of Figure 14A and B in the text.

ASb-2 (1 h)

Control	ASb-2 (50 µM)	CCCP (40 µM)	CCCP+ASb-2
103.649	24.568	110.757	89.703
98.919	18.703	109.108	85.405
97.432	24.189	111.730	87.865
100.000	22.486	110.532	87.658
3.246	3.282	1.325	2.156

ASb-2 (3 h)

Control	ASb-2 (50 µM)	CCCP (40 µM)	CCCP+ASb-2
112.825	12.568	72.035	37.898
93.443	12.703	70.588	33.558
93.732	14.189	59.016	24.879
99.9999	13.15315	67.21305	32.11183
11.1081	0.899774	7.135311	6.628599

ASb-2 (6 h)

Control	ASb-2 (50 µM)	CCCP (40 µM)	CCCP+ASb-2
109.894	50.000	19.858	5.010
98.587	45.390	15.957	5.958
90.459	36.170	14.539	6.093
99.64664	43.85343	16.7849	5.6872
9.760585	7.041783	2.75442	0.5902

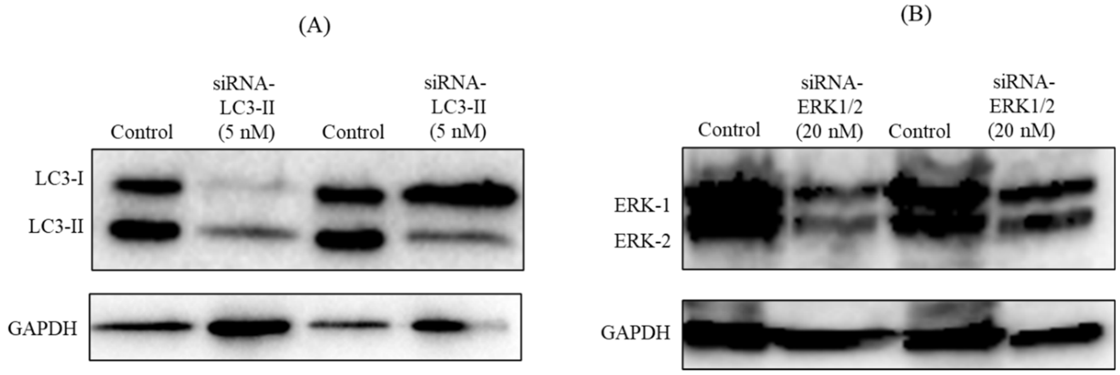

Figure S1. Knock-down of LC3-II and ERK1/2 by the corresponding siRNAs (Western blot analysis). A) Western blot results of LC3-II knock-down by siRNA against LC3-II in Jurkat cells. B) Western blot results of ERK1/2 knock-down by siRNA against ERK1/2 in Jurkat cells.
